# The dCache Chemoreceptor TlpA of Helicobacter pylori Binds Multiple Attractant and Antagonistic Ligands via Distinct Sites

**DOI:** 10.1128/mBio.01819-21

**Published:** 2021-08-03

**Authors:** Kevin S. Johnson, Bassam A. Elgamoudi, Freda E.-C. Jen, Christopher J. Day, Emily Goers Sweeney, Megan L. Pryce, Karen Guillemin, Thomas Haselhorst, Victoria Korolik, Karen M. Ottemann

**Affiliations:** a Department of Microbiology and Environmental Toxicology, University of California, Santa Cruz, California, USA; b Institute for Glycomics, Griffith University, Gold Coast Campus, Southport, QLD, Australia; c Institute of Molecular Biology, University of Oregon, Eugene, Oregon, USA; University of Utah

**Keywords:** *Helicobacter pylori*, chemotaxis, ligand discovery, dCache, signal transduction, chemoreceptor, inflammation, receptor-ligand interaction

## Abstract

The Helicobacter pylori chemoreceptor TlpA plays a role in dampening host inflammation during chronic stomach colonization. TlpA has a periplasmic dCache_1 domain, a structure that is capable of sensing many ligands; however, the only characterized TlpA signals are arginine, bicarbonate, and acid. To increase our understanding of TlpA’s sensing profile, we screened for diverse TlpA ligands using ligand binding arrays. TlpA bound seven ligands with affinities in the low- to middle-micromolar ranges. Three of these ligands, arginine, fumarate, and cysteine, were TlpA-dependent chemoattractants, while the others elicited no response. Molecular docking experiments, site-directed point mutants, and competition surface plasmon resonance binding assays suggested that TlpA binds ligands via both the membrane-distal and -proximal dCache_1 binding pockets. Surprisingly, one of the nonactive ligands, glucosamine, acted as a chemotaxis antagonist, preventing the chemotaxis response to chemoattractant ligands, and acted to block the binding of ligands irrespective of whether they bound the membrane-distal or -proximal dCache_1 subdomains. In total, these results suggest that TlpA senses multiple attractant ligands as well as antagonist ones, an emerging theme in chemotaxis systems.

## INTRODUCTION

Chemotaxis is a vital host colonization strategy used by many pathogens, including Helicobacter pylori, Campylobacter jejuni, Borrelia burgdorferi, Pseudomonas aeruginosa, Vibrio cholerae, and Salmonella enterica. How chemotaxis benefits bacteria, however, varies. Pathogens have been found to use chemotaxis to access growth-promoting nutrients, locate signaling molecules that regulate virulence gene expression, spread throughout tissues and into specific niches, and affect host interactions that control inflammation (1). Chemotaxis signaling systems are highly conserved, and their widespread presence in pathogens underscores the importance of understanding their roles in colonization (1, 2).

One pathogen that requires chemotaxis for multiple infection aspects is H. pylori. This Gram-negative bacterium chronically colonizes the stomach of nearly half of the world’s population and ∼35% of individuals in the United States (3). Stomach colonization results in chronic inflammation, and a subset of individuals develop ulcers and gastric cancer (4, 5). H. pylori presents a significant disease burden, with ∼700,000 deaths from gastric cancer yearly (6). While many people are infected, the degree of host inflammation varies, which ultimately drives disease severity (7–9). We understand some H. pylori properties that dictate inflammation severity, such as the Cag pathogenicity island (10, 11). Still, the full compendium of H. pylori properties that modulate this host response is not yet understood.

H. pylori chemotaxis has been linked to host inflammation (12–15). Specifically, mutants missing key chemotaxis signal transduction proteins trigger less host inflammation despite achieving normal colonization levels, while mutants missing either the chemoreceptor TlpA or TlpB cause elevated inflammation (13–15). Chemoreceptors head the chemotaxis signal transduction system and dictate which signals a bacterium responds to. The loss of individual chemoreceptors within a system alters a bacterium’s sensing profile but does not cause a complete loss of chemotactic ability, presumably biasing the bacterium toward signals sensed by the remaining chemoreceptors.

H. pylori possesses four chemoreceptors: TlpA, TlpB, TlpC, and TlpD. Each of these plays nonidentical roles in infection. TlpA, -C, and -D are required for colonization, while TlpA and -B are required for inflammation control (15–17). In this work, we focus on TlpA, which plays multiple roles in promoting early but not late colonization and dampening later inflammation. At early times, 2 weeks postinfection, H. pylori Δ*tlpA* displays a modest colonization defect compared to the wild type (WT) as the sole infecting strain, a deficiency exacerbated by WT coinfection (15–17). However, during the chronic stage of infection after 6 months, H. pylori Δ*tlpA* bacteria colonize to normal levels but induce significantly more histologically evident inflammation than the WT (15).

TlpA is a transmembrane chemoreceptor with a periplasmic double-Cache (dCache_1) ligand binding domain (LBD) (18, 19). Cache domains are ubiquitous extracellular sensing domains found in both eukaryotes and prokaryotes, where they are the most common extracellular sensing domains (18). Cache domains bind a wide variety of small molecules but mostly amino acids, modified amino acids, and carboxylic acids (18). Many Cache domains have been found to bind multiple ligands (18). dCache_1 domains have two Cache subdomains, a membrane-distal and -proximal subdomain, each of which can bind ligands, although most commonly, the ligands are bound in the membrane-distal domain (18, 20, 21). TlpA has some identified chemotaxis-active ligands, including arginine and sodium bicarbonate (22, 23). Additionally, TlpA has been shown to play a subtle role in sensing acidic pH but to a much lesser extent than TlpB or TlpD (24). Whether TlpA senses additional ligands or how any of these ligands are bound, however, is unknown.

Given the sensing potential of dCache_1 chemoreceptors, we hypothesized that TlpA would be capable of sensing ligands beyond those previously reported. Knowing a full set of ligands is critical for interpreting the TlpA-associated phenotypes. In this study, we identified new TlpA ligands and characterized their binding and ability to induce a chemotactic response. Ligand binding arrays were used to screen a broad set of ligands for binding to TlpA, resulting in the identification and verification of seven TlpA-specific ligands. The use of a temporal chemotaxis assay enabled us to determine that three ligands, arginine, fumarate, and cysteine, acted as TlpA-sensed chemoattractants, while the other ligands elicited no response. Molecular modeling experiments, assessment of TlpA point mutants, and surface plasmon resonance (SPR) competition assays suggested that TlpA ligands interact with two distinct sites. Furthermore, one of the high-affinity nonchemotactic TlpA ligands, glucosamine, blocked attractant responses to, and binding of, chemoattractant ligands, thus acting as an antagonist. Overall, our findings suggest that TlpA responds to several key H. pylori nutrients using both dCache_1 subdomains, with some acting as agonists and some acting as antagonists for a chemotaxis attractant response.

## RESULTS

### TlpA interacts directly with multiple ligands.

TlpA’s LBD (TlpA_LBD_) interactions with potential ligands were assessed using small-molecule arrays containing amino acids, organic acids, salts, and glycans (see Table S1 in the supplemental material). TlpA_LBD_ bound seven small molecules: arginine, cysteine, fumarate, glucosamine, malic acid, thiamine, and α-ketoglutarate (Table 1 and Fig. S1). Glycan arrays containing both simple and complex glycans were also interrogated, but no binding was detected.

**TABLE 1:** TlpA_LBD_ ligand binding analysis from the ligand binding array screen and surface plasmon resonance^*a*^

Ligand	Array result	Mean binding affinity (μM) ± SD
Arginine	+	2 ± 0.11
Cysteine	+/−	4.7 ± 0.3
Fumarate	+	10 ± 1.53
Glucosamine	+	10.5 ± 2.8
Malic acid	+/−	46 ± 17
Thiamine	+/−	60 ± 0.6
α-Ketoglutarate	+	224 ± 11.2

a Data represent the mean values ± SD from three independent experiments (*n* = 3). For the ligand binding array, results are reported as + for positive binding, +/− for intermediate binding, and − for no binding. Binding affinity (micromolar) was determined by SPR.

10.1128/mBio.01819-21.1 FIG S1 Representative sensorgrams from surface plasmon resonance (SPR) analysis of TlpA_LBD_ with arginine (A), cysteine (B), fumarate (C), glucosamine (D), malic acid (E), thiamine (F), and alpha-ketoglutarate (G). Download FIG S1, PDF file, 0.8 MB. Copyright © 2021 Johnson et al. 2021 Johnson et al. https://creativecommons.org/licenses/by/4.0/ This content is distributed under the terms of the Creative Commons Attribution 4.0 International license.

10.1128/mBio.01819-21.5 TABLE S1 Amino acids, salts of organic acids, other chemotactic small molecules, and glycans printed on the small-molecule chemotaxis arrays. Download Table S1, PDF file, 1.4 MB. Copyright © 2021 Johnson et al. 2021 Johnson et al. https://creativecommons.org/licenses/by/4.0/ This content is distributed under the terms of the Creative Commons Attribution 4.0 International license.

We next determined TlpA_LBD_ ligand binding affinities by surface plasmon resonance (SPR). Arginine, cysteine, fumarate, and glucosamine all exhibited high-affinity binding (dissociation constant [*K_d_*] of <10 μM), while malic acid, thiamine, and α-ketoglutarate showed lower affinities (>45 μM) (Table 1). Overall, these data suggest that the TlpA_LBD_ can interact directly with these seven ligands, with affinities ranging from 2 to 224 μM.

### Some TlpA ligands act as chemoattractants, while others elicit no response.

**FIG 1:**
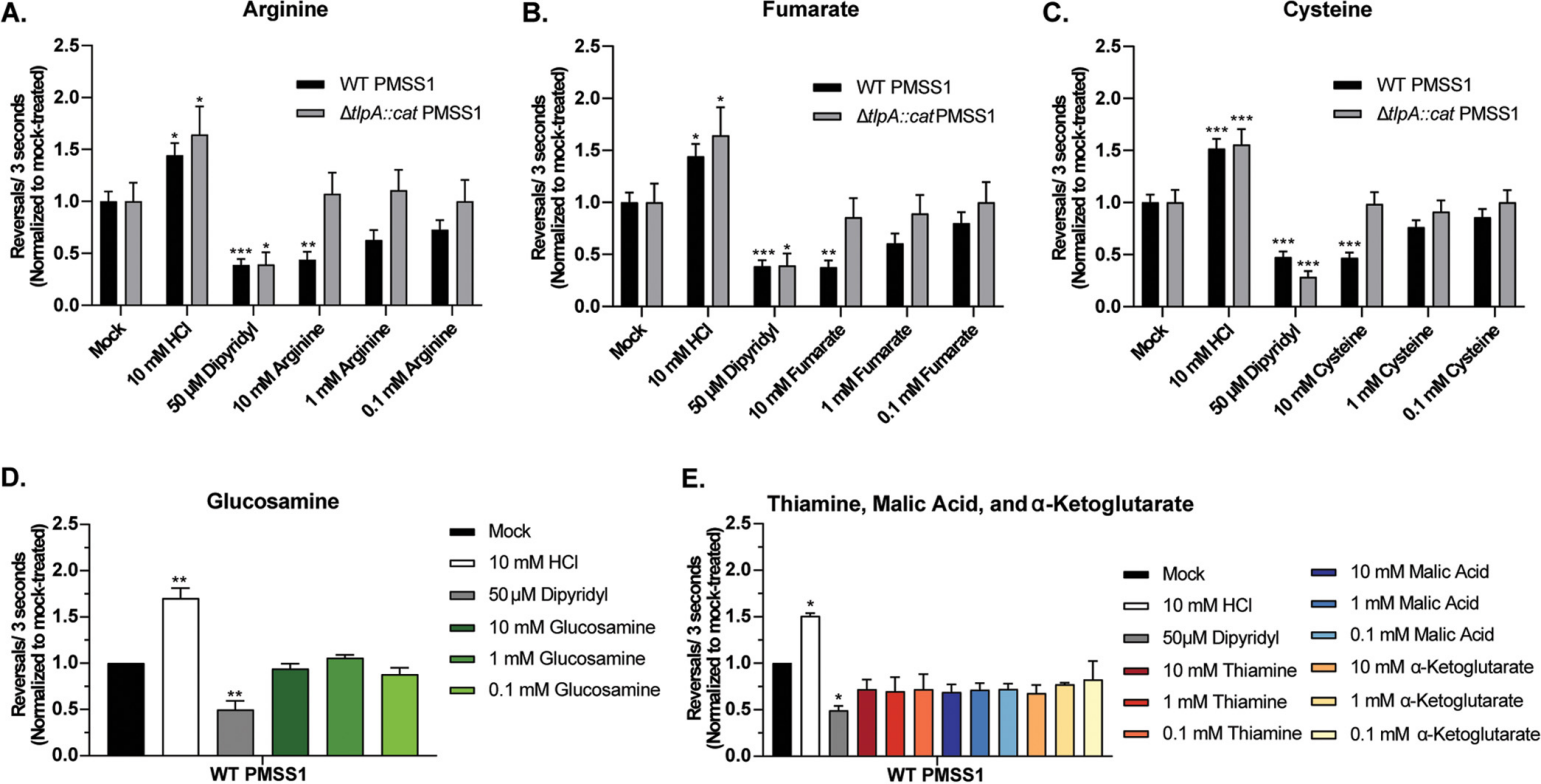
H. pylori responds to arginine, fumarate, and cysteine as TlpA-dependent chemoattractants in the temporal chemotaxis assay. Cultures of H. pylori PMSS1 WT and Δ*tlpA* strains were grown overnight, back-diluted, and then incubated until an OD_600_ of 0.12 to 0.15 was reached. Cultures were treated with water (Mock) or various concentrations of compounds, as indicated. For panels C and E, the pH of cysteine, thiamine, malic acid, and α-ketoglutarate stocks was adjusted using NaOH to match the pH of the water used for the untreated control. The cells were immediately filmed, and direction changes were counted over a 3-s swimming period in at least 100 cells per treatment from 3 biological replicates. Data are normalized to the values for the untreated control for each strain, as described in Materials and Methods. Error bars represent the standard errors of the means. *, *P* < 0.05; **, *P* < 0.01; ***, *P* < 0.001 (comparisons to the untreated control per strain using two-way ANOVA with Dunnett’s multiple-comparison test).

H. pylori chemotactic responses toward the seven putative TlpA-dependent ligands were examined by a live-cell video microscopy assay that measures the temporal chemotaxis response to test ligands. In this assay, attractants elicited fewer direction changes, and repellents elicited more direction changes, compared to basal levels (20, 25–30). Several TlpA ligands were acidic in solution, leading to the appearance of significant TlpA-independent chemorepellent responses; these were cysteine, thiamine, malic acid, and α-ketoglutarate (Fig. S2A to C). Acidic conditions are sensed by chemoreceptors other than TlpA (24, 29, 31) and potentially mask chemotactic responses to the ligands being tested. Accordingly, the pH of the ligand stocks for cysteine, thiamine, malic acid, and α-ketoglutarate was neutralized using NaOH to match the pH of the water used in the mock-treated control. This treatment eliminated the confounding effect of medium acidification when assessing chemotactic responses (Fig. S2C). After incorporating these adjustments, we found that the addition of arginine, fumarate, or cysteine resulted in fewer direction changes for WT H. pylori (Fig. 1A to C), suggesting that these compounds were attractants. The highest concentration tested, 10 mM, induced the most significant and robust attractant responses for each ligand (arginine, *P* < 0.01; fumarate, *P* < 0.01; cysteine, *P* < 0.001) (Fig. 1A to C). Responses to 1 and 0.1 mM ligands were apparent but not significant compared to the untreated control. Glucosamine, thiamine, malic acid, and α-ketoglutarate induced no significant direction changes at any concentration tested (Fig. 1D and E), suggesting that they do not act as attractants or repellents.

10.1128/mBio.01819-21.2 FIG S2 Thiamine, alpha-ketoglutarate, cysteine, and malic acid trigger TlpA-independent repellent responses. (A and B) Cultures of WT and Δ*tlpA*::*cat* PMSS1 were grown in BB10 overnight and then treated with various concentrations of thiamine or alpha-ketoglutarate. (C) Cultures of WT PMSS1 were grown as described above and treated with 10 mM pH neutralized or unadjusted thiamine, alpha-ketoglutarate, cysteine, and malic acid. For all experiments, cells were immediately filmed after ligand addition, and direction changes were counted over a 3-second swimming period in at least 100 cells per treatment. Repellents cause increases in direction changes, as exemplified by the control repellent HCl, while attractants cause fewer direction changes, as exemplified by the control attractant dipyridyl. Error bars represent the standard error of the mean. *, *P* < 0.05; **, *P* < 0.01, comparisons to untreated control per strain using two-way ANOVA, Dunnett’s multiple comparison test. Download FIG S2, PDF file, 0.6 MB. Copyright © 2021 Johnson et al. 2021 Johnson et al. https://creativecommons.org/licenses/by/4.0/ This content is distributed under the terms of the Creative Commons Attribution 4.0 International license.

To determine if chemoattractant responses toward arginine, fumarate, and cysteine were TlpA dependent, the same tracking experiments were repeated with a mutant lacking *tlpA* (Δ*tlpA*). The Δ*tlpA* mutant retained general chemotactic ability, producing significant attractant and repellent responses to the controls dipyridyl and HCl, respectively, but failed to exhibit a chemotactic response to arginine, fumarate, or cysteine (Fig. 1A to C). These results suggest that the high-affinity ligands arginine, fumarate, and cysteine are TlpA-dependent chemoattractants.

### TlpA has at least two ligand interaction sites.

**FIG 2:**
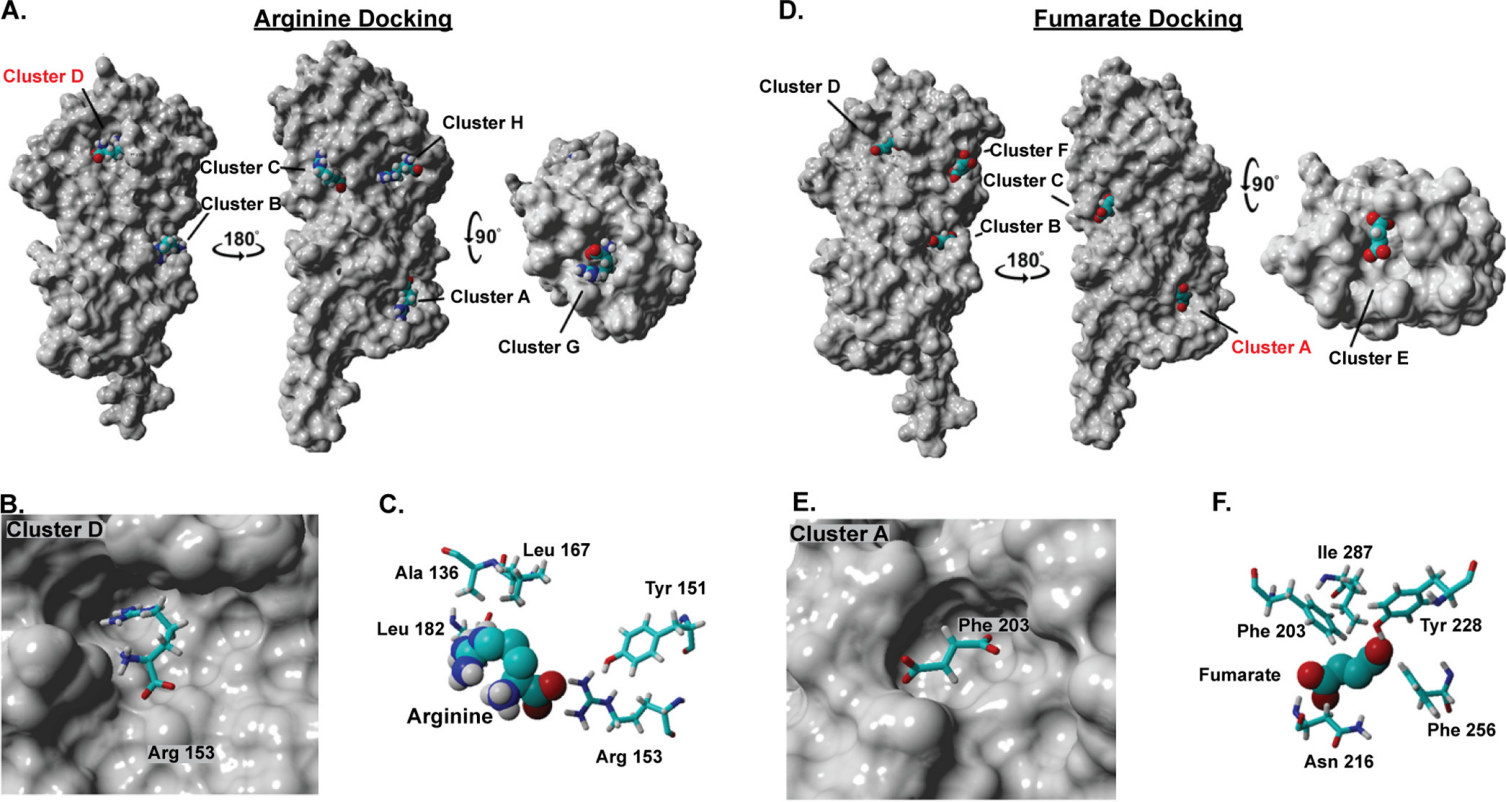
Docking analysis of TlpA and arginine or fumarate identifies several clusters that are occupied by these ligands. (A and D) Docking analysis shows several clusters occupied by arginine (A) and fumarate (D) on the surface of a space-filling version of TlpA_LBD_. Clusters C and G are biologically irrelevant due to the homodimer formation of TlpA, and clusters B and H are poorly populated (see Table S3 in the supplemental material). (B and C) View of the energetically preferred bound conformation of arginine in cluster D, the predicted membrane-distal dCache pocket, with key interacting amino acids shown. (E and F) View of the energetically preferred bound conformation of fumarate in cluster A, the membrane-proximal dCache pocket, with key interacting amino acids shown.

To gain insight into how TlpA_LBD_ binds ligands, a blind docking modeling experiment was carried out using AutoDock Vina (32, 33). We focused on two high-affinity ligands, arginine and fumarate. We found that these two ligands occupied two main sites, referred to as clusters (Table S2), which map to the membrane-distal and -proximal dCache_1 binding pockets (Fig. 2A and D). Arginine was placed mostly in cluster D (55%) (Table S3), which is located in the membrane-distal dCache_1 domain. Arg153 dominated this binding interaction, with stabilization from Tyr151 (Fig. 2B and C). Fumarate, in contrast, was placed mostly in cluster A (45%) (Table S3) in the membrane-proximal dCache_1 domain, with Phe203 being the most crucial residue required for interaction with fumarate (Fig. 2E and F). The docking experiment further revealed that although fumarate and arginine are likely to have two distinct preferred binding sites, they both can bind to their reciprocal sites. For example, 25% of the models had arginine found in fumarate’s preferred cluster A (Table S3). Overall, these analyses suggest that arginine is more likely to bind the membrane-distal dCache_1 domain, while fumarate is more likely to bind the membrane-proximal dCache_1 domain, but binding to the other binding pockets is also possible.

10.1128/mBio.01819-21.6 TABLE S2 Clusters A to H and their interacting amino acids. Download Table S2, PDF file, 0.5 MB. Copyright © 2021 Johnson et al. 2021 Johnson et al. https://creativecommons.org/licenses/by/4.0/ This content is distributed under the terms of the Creative Commons Attribution 4.0 International license.

10.1128/mBio.01819-21.7 TABLE S3 TlpA_LBD_ docking analysis with fumarate and arginine. Download Table S3, PDF file, 0.4 MB. Copyright © 2021 Johnson et al. 2021 Johnson et al. https://creativecommons.org/licenses/by/4.0/ This content is distributed under the terms of the Creative Commons Attribution 4.0 International license.

**FIG 3:**
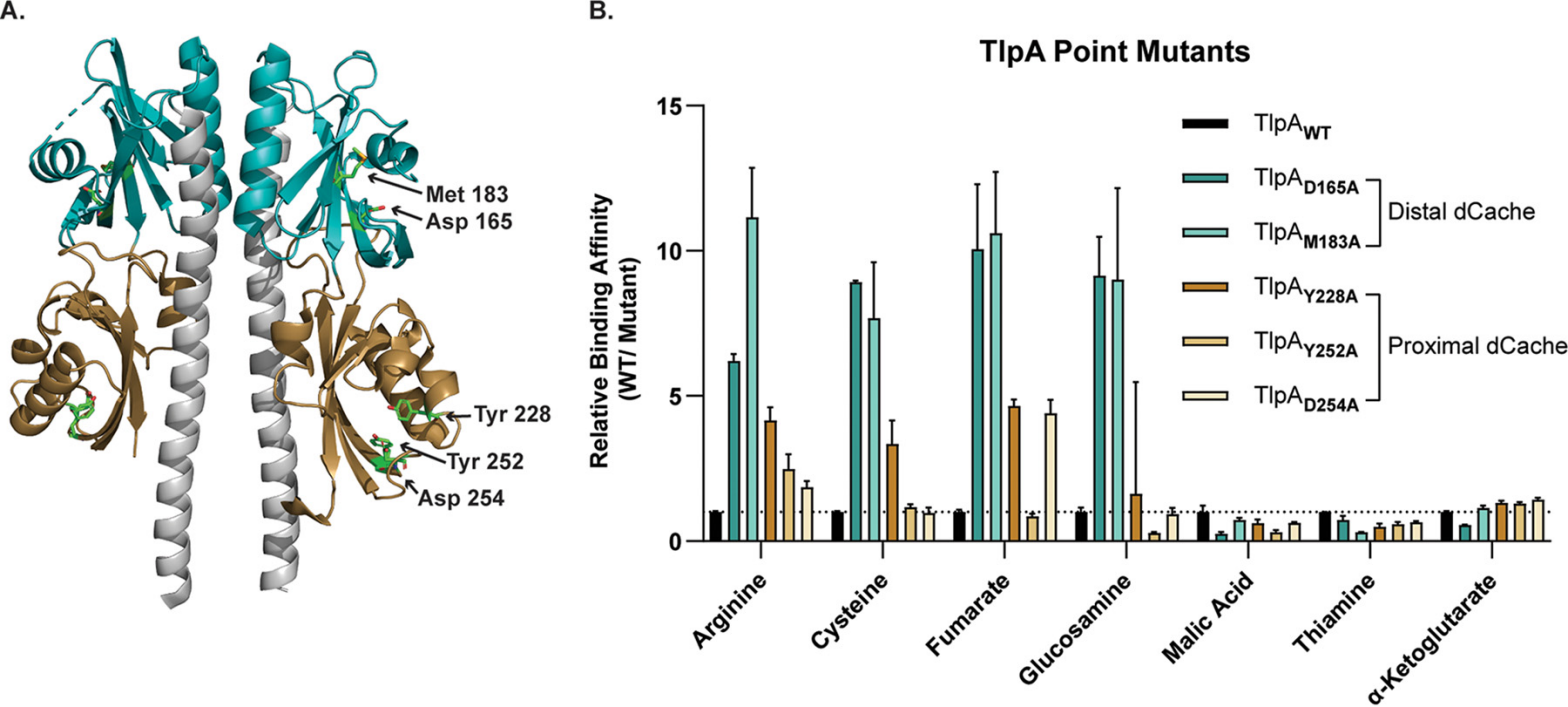
TlpA_LBD_ binds chemotaxis-active ligands through the membrane-distal or -proximal dCache subdomains. (A) Ribbon diagram of TlpA_LBD_ as a homodimer. The membrane-distal and membrane-proximal dCache domains are shown in teal and gold, respectively. Residues that were mutated to alanine are highlighted in each region to make TlpA_D165A_, TlpA_M183A_, TlpA_Y228A_, TlpA_Y252A_, and TlpA_D254A_. (B) Relative binding affinities of each ligand for the WT and TlpA_LBD_ membrane-distal and -proximal dCache point mutants. For example, mutation of D165 to A resulted in an ∼6-fold decrease in the arginine binding affinity compared to WT binding. Data represent the mean values and standard errors of the means from three independent experiments (*n* = 3).

To further study TlpA-ligand interactions, we generated TlpA_LBD_ point mutants at residues in the membrane-distal (D165A and M183A) or membrane-proximal (Y228A, Y252A, and D254A) binding pockets and determined the binding affinity of the resultant proteins for all ligands (Table 2 and Fig. 3A). Mutation of either membrane-distal residue resulted in an ∼10-fold decrease in the binding affinity for arginine, cysteine, fumarate, and glucosamine (Fig. 3B and Table 2). Mutation of the membrane-proximal residue TlpA_Y228A_ also led to a decrease in the binding affinity for arginine, cysteine, and fumarate, but it was only 4-fold. The other mutation in the membrane-proximal site (TlpA_D254A_) affected only fumarate binding (Fig. 3B and Table 2). No proximal pocket residues affected the binding affinity of glucosamine. Membrane-distal and -proximal mutations resulted in a modest ∼3- to 4-fold increase in the binding affinity for malic acid and thiamine compared to the WT control. Additionally, no appreciable change in the binding affinity for α-ketoglutarate was observed for any point mutant. Overall, TlpA binding interactions by the high-affinity ligands arginine, cysteine, fumarate, and glucosamine are most disrupted by mutations in the membrane-distal dCache_1 domain, but mutations in the membrane-proximal dCache_1 also significantly impair fumarate and, to a lesser extent, arginine and cysteine binding, consistent with the predictions from the docking analysis.

**TABLE 2:** Binding affinities of TlpA_LBD_ and TlpA_LBD_ membrane-distal and -proximal dCache mutants for TlpA ligands^*a*^

Ligand	Mean binding affinity (μM) ± SD					
	TlpA_WT_	TlpA_D165A_	TlpA_M183A_	TlpA_Y228A_	TlpA_Y252A_	TlpA_D254A_
Arginine	2 ± 0.11	12.4 ± 0.84	22.3 ± 5.9	8.3 ± 1.6	4.96 ± 1.8	3.71 ± 0.73
Cysteine	4.7 ± 0.3	41.9 ± 0.4	36.1 ± 15.7	15.7 ± 6.6	5.5 ± 0.8	4.58 ± 1.5
Fumarate	10 ± 1.5	100.5 ± 38.9	106.1 ± 36.6	46.6 ± 3.9	8.5 ± 1.7	44 ± 8.1
Glucosamine	10.5 ± 2.8	96 ± 24.3	94.6 ± 57.3	17.1 ± 70	2.96 ± 0.7	9.84 ± 3.7
Malic acid	46 ± 17	11.9 ± 4.3	33.6 ± 6.2	28.6 ± 9.4	14.2 ± 6.2	29.3 ± 2
Thiamine	60 ± 0.6	43.9 ± 14.1	18.1 ± 1.2	29.5 ± 12.3	35 ± 7.3	39.3 ± 4.3
α-Ketoglutarate	224 ± 11.2	124 ± 7.1	256.2 ± 27.6	295.4 ± 27.7	289 ± 22.6	321 ± 21.6

a Data represent the mean values ± SD from three independent experiments (*n* = 3).

### TlpA_LBD_ binds arginine and fumarate through distinct binding sites.

The above-described data suggest that TlpA_LBD_ can bind ligands in both dCache_1 subdomain binding pockets. To further analyze the possibility of two distinct binding sites for chemotaxis-active ligands in TlpA_LBD_, we employed a competition SPR (A-B-A) binding assay focused on arginine and fumarate because they were both chemoattractants and predicted to bind different sites preferentially. In this assay, the competition for binding to TlpA_LBD_ between arginine and fumarate is assessed by adding the ligands sequentially and monitoring whether the SPR signal changes upon the addition of the second ligand. The two ligands’ binding status can be classified as either independent, shared, or preferential shared sites. For independent sites, ligand A saturates all its binding sites, and ligand B then binds to its independent site; this mode produces additive effects on the SPR signal. Shared sites, in contrast, do not produce additive/cumulative effects; i.e., ligand A binds its site and then blocks ligand B from the same site. Finally, it is also possible to have preferential shared sites where ligands share the same binding site, but the protein binds to one ligand preferentially when in equilibrium.

**FIG 4:**
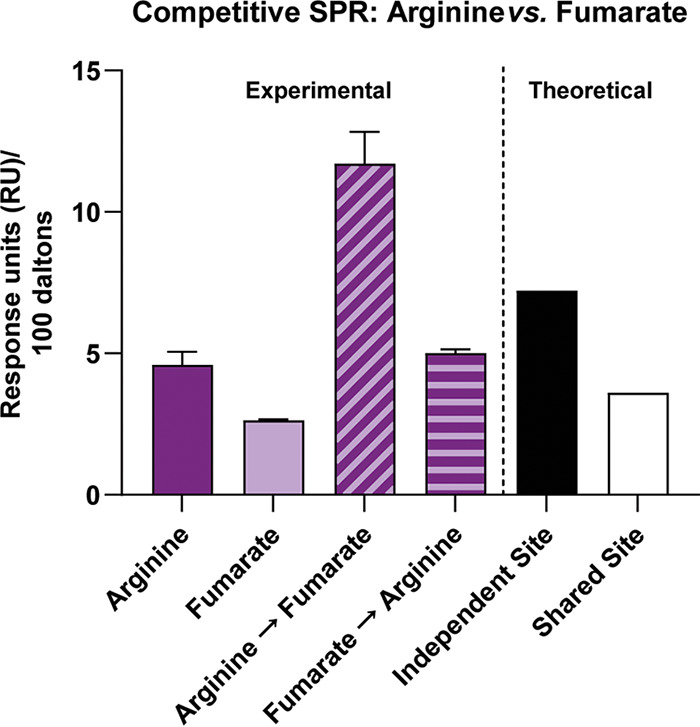
SPR competition analysis indicates the presence of two distinct binding sites for TlpA ligands. SPR competition analyses of binding by arginine and fumarate to WT TlpA_LBD_ were performed. Compounds were used at concentrations 10-fold higher than their respective *K_d_* values. Arginine, response to arginine only; Fumarate, response to fumarate only; Arginine→Fumarate, fumarate response after saturation with arginine; Fumarate→Arginine, arginine response after saturation with fumarate. The theoretical values are responses units based on mathematical theory: independent site is the sum of individual responses, and shared site is the sum of individual responses divided by the number of individual responses. All response data were normalized to a molecular weight of 100 Da for each analyte, allowing direct comparison of responses.

We first saturated TlpA_LBD_ with arginine and then added fumarate. In this case, an increased response (additive effect) was observed, compared to the theoretical value (Fig. 4). This outcome suggests that fumarate and arginine bind to independent sites. Conversely, when TlpA_LBD_ was saturated with fumarate, arginine did not produce an additional response, compared to the theoretical value (Fig. 4). This result suggests that fumarate prevented arginine binding because either arginine competed with fumarate at the same site(s) or fumarate caused an allosteric effect that prevents arginine binding, a common occurrence in sensory proteins (34). Overall, the docking and competitive SPR data support the hypothesis that there are two binding sites with possible cooperative interactions or overlap between them.

**FIG 5:**
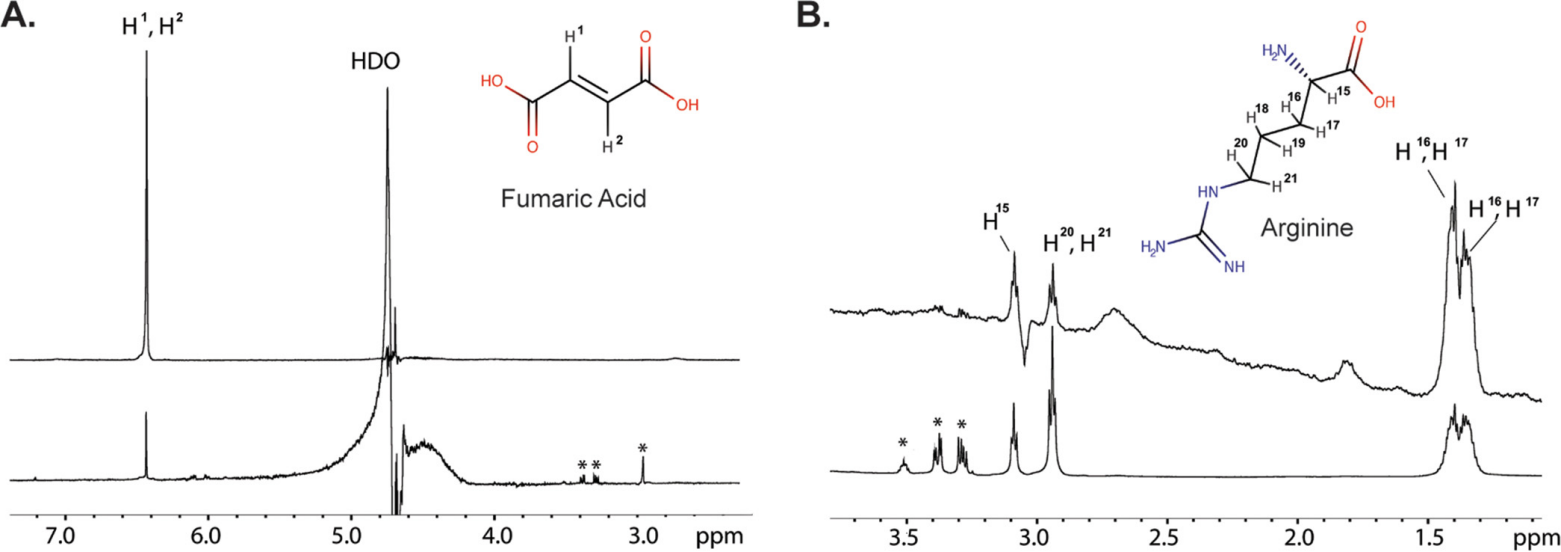
STD NMR analysis supports that TlpA_LBD_ binds fumarate and arginine. ^1^H NMR spectra are shown at the bottom for fumarate (A) and arginine (B). The STD NMR spectra are shown at the top, acquired at 600 MHz at 289 K, with an on-resonance of −1 ppm, an off-resonance of 33 ppm, and a total saturation time of 2 s.

The docking analysis and competition SPR assay suggested that arginine and fumarate bind to distinct TlpA_LBD_ sites; therefore, we sought to further characterize TlpA-ligand interactions using saturation transfer difference (STD) nuclear magnetic resonance (NMR) spectroscopy, which can measure protein-ligand interactions and ascertain which part of a ligand interacts with the receptor protein (35, 36). When TlpA_LBD_ bound fumarate, a significant STD NMR signal was detected, consistent with the two ethylene protons interacting with the protein (Fig. 5A). Similarly, when TlpA_LBD_ bound arginine, significant STD NMR signals were observed (Fig. 5B). On arginine, the relative STD NMR effects showed that the H-3 and H-4 protons on the side chain received the largest saturation transfer from the protein protons, indicating that arginine interacts with TlpA_LBD_ around its middle carbon side chain region (Fig. 5B). These results therefore provide additional confirmation that TlpA interacts with fumarate and arginine, mostly along the carbon chain backbones in each ligand.

### A non-chemotaxis-active TlpA ligand can antagonize chemoattractant responses.

**FIG 6:**
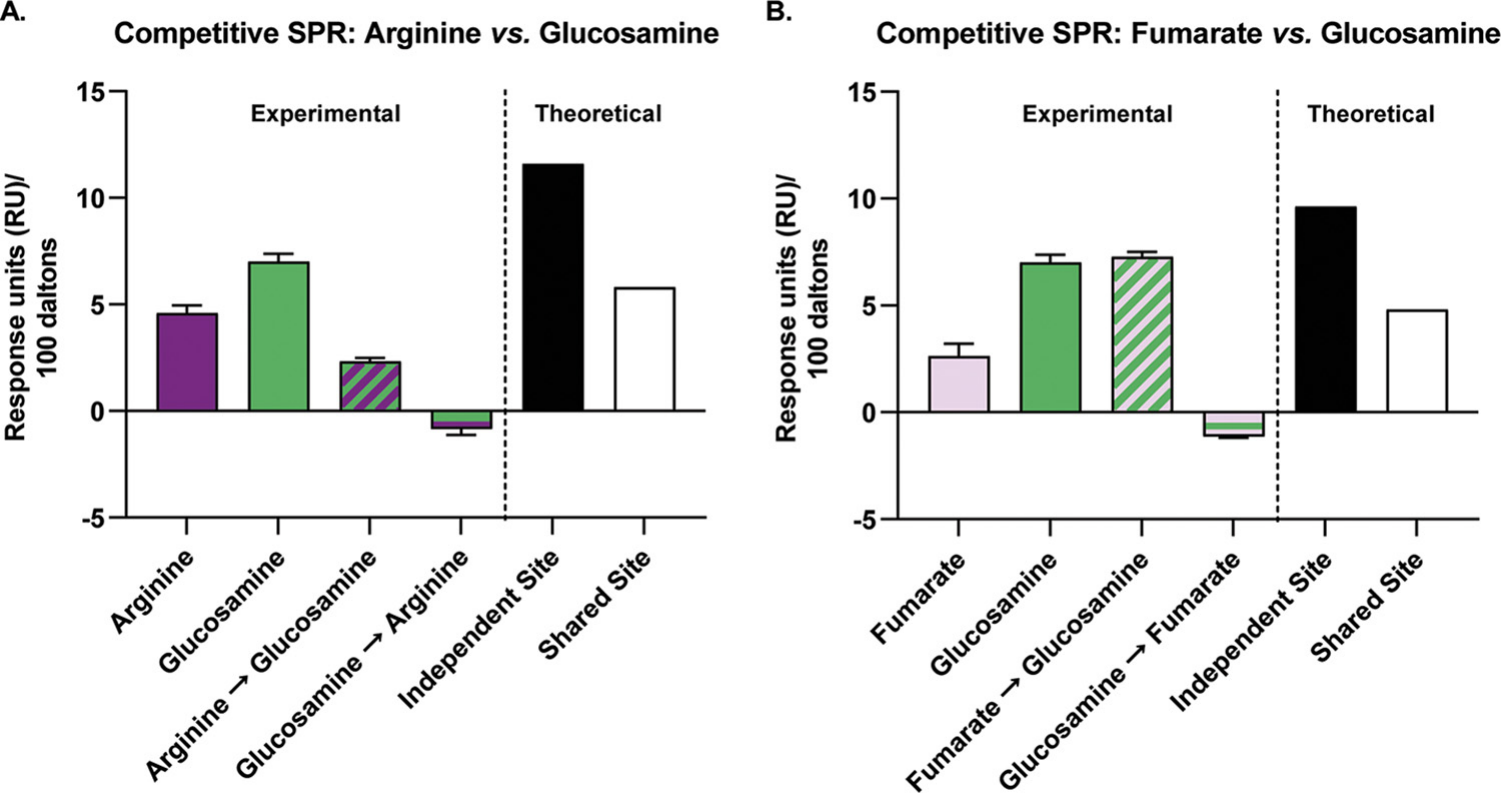
SPR competition analysis demonstrates that glucosamine blocks the binding of TlpA chemoattractants. Data from SPR competition analysis of binding of arginine, fumarate, and glucosamine to WT TlpA_LBD_ are shown. Compounds were used at concentrations 10-fold higher than their respective *K_d_* values. Arginine, response to arginine only; Glucosamine, response to glucosamine only; Arginine→Glucosamine, response to glucosamine following saturation with arginine; Glucosamine→Arginine, response to arginine following saturation with glucosamine; Fumarate, response to fumarate only; Fumarate→Glucosamine, response to glucosamine following saturation with fumarate; Glucosamine→Fumarate, response to fumarate following saturation with glucosamine. The theoretical values are response unit values based on mathematical theory. All response data were normalized to a molecular weight of 100 Da for each analyte, allowing direct comparison of responses.

It was surprising to find a high-affinity direct binding ligand, glucosamine, that bound to the membrane-distal dCache_1 subdomain (Fig. 3) and did not elicit a chemotaxis response (Fig. 1D). Previous reports on ligand interactions with chemoreceptors in Escherichia coli and P. aeruginosa suggested that some ligands bind chemoreceptors as antagonists, blocking normal chemotactic responses toward chemotaxis-active ligands (37, 38). Consequently, we tested whether glucosamine could block the binding of the chemotaxis-active TlpA ligands arginine and fumarate using a competitive SPR assay. Of note, the other nonchemoactive TlpA ligands, malic acid, thiamine, and α-ketoglutarate, were not affected by either membrane-proximal or -distal dCache_1 domain point mutants (Fig. 3); therefore, we hypothesized that they would be unable to affect the binding of chemotaxis-active TlpA ligands, as they do not appear to bind through the same sites. The results showed that when glucosamine was added following saturation with arginine or fumarate, the response was not additive, suggesting that glucosamine competed with both arginine and fumarate (Fig. 6A and B). However, when arginine or fumarate was added to TlpA_LBD_ following initial saturation with glucosamine, no additive response was observed (Fig. 6A and B). This result suggests that glucosamine can prevent the binding of both ligands to TlpA_LBD_.

**FIG 7:**
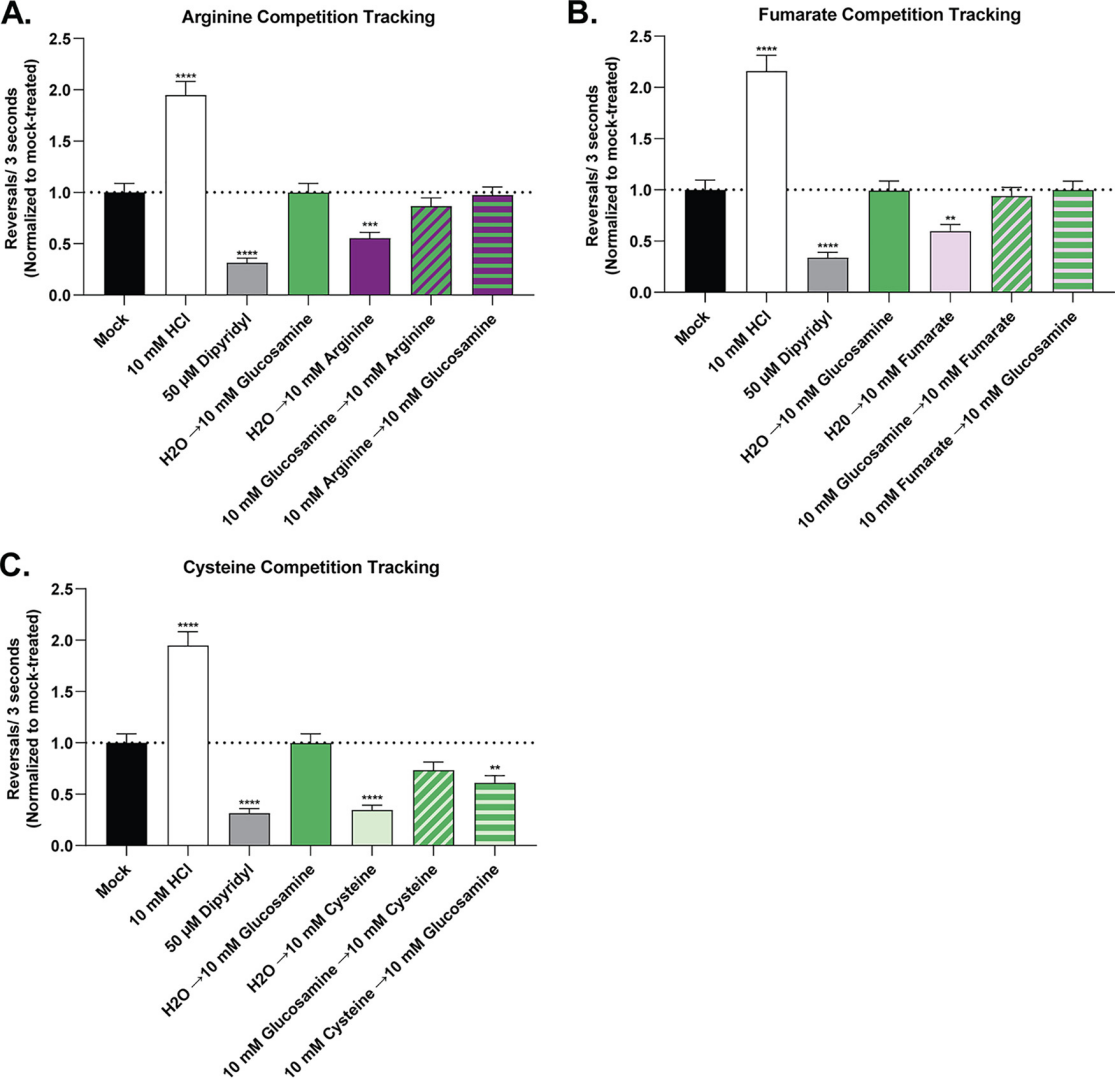
Ligand competition tracking experiment between chemoactive and nonchemoactive TlpA ligands. Cultures of the H. pylori PMSS1 WT were grown in BB10 overnight and then back-diluted as described in the legend of Fig. 1. Cultures were mock treated or treated with various concentrations of compounds as indicated. The pH of the cysteine stock was adjusted using NaOH to match the pH of the water used for the untreated control. The cells were immediately filmed, and direction changes were counted over a 3-s swimming period in at least 100 cells per treatment from 3 biological replicates. Repellents increase direction changes, as exemplified by the control repellent HCl, while attractants decrease direction changes, as exemplified by the control attractant dipyridyl. Data are normalized to the values for the untreated control for each strain, as described in Materials and Methods. Error bars represent the standard errors of the means. *, *P* < 0.05; **, *P* < 0.01; ***, *P* < 0.001 (comparisons to the untreated control per strain using two-way ANOVA and Dunnett’s multiple-comparison test).

Consequently, we tested whether glucosamine affected H. pylori chemotaxis by developing a ligand competition tracking assay between non-chemotaxis-active and chemotaxis-active TlpA ligands. This assay is a modified version of our live-cell video microscopy assay where the addition of a chemotaxis-active ligand is followed by the addition of a non-chemotaxis-active ligand 10 s later and vice versa. Using this approach, we determined that glucosamine addition prevented the chemoattractant response to arginine and fumarate and severely blunted the response to cysteine (Fig. 7). This response was decreased regardless of whether glucosamine was added before or after the addition of the chemoattractant. In total, these results suggest that glucosamine blocks chemotaxis-active ligand binding and acts as a TlpA chemotaxis antagonist.

## DISCUSSION

We report the identification of several ligands specific for the H. pylori dCache_1 chemoreceptor TlpA, including confirmation of previous reports that TlpA interacts with arginine (22, 23). Arginine, along with fumarate and cysteine, functioned as a TlpA-sensed chemotaxis attractant. Furthermore, these chemotaxis-active ligands appeared to interact with the membrane-distal and -proximal dCache_1 domains. Finally, we found that glucosamine acts as a chemotaxis antagonist, blocking TlpA binding and responses to multiple attractants.

### TlpA can bind a broad set of ligands with diverse biological functions.

TlpA bound a broad set of molecules ranging from the amino acid arginine, with a large, charged side chain; to the amino acid cysteine, with a smaller, polar side chain; organic acids, including fumarate, malic acid, and α-ketoglutarate; the large vitamin thiamine; and the amino sugar glucosamine. Ultimately, our data suggest that only some of these ligands bind within the canonical dCache_1 binding pockets, including all those that affected chemotaxis. Thus, these results agree with previous reports showing that individual dCache chemoreceptors can sense diverse types of ligands (39, 40).

The three molecules that elicited a chemotaxis response, arginine, fumarate, and cysteine, have been shown to have important biological roles in H. pylori biology. Arginine is an essential amino acid for H. pylori under *in vitro* growth conditions (41), and H. pylori uses arginine to promote acid tolerance and dampen host immune responses (42–45). Fumarate is predicted to be an alternative terminal electron acceptor for growth under anaerobic respiration (46), and the associated enzyme, fumarate reductase, is essential for H. pylori colonization *in vivo* (47). Additionally, fumarate is highly depleted when H. pylori is cocultured with gastric organoids, consistent with the prediction that it is preferentially used *in vivo* (48). Finally, cysteine is an essential amino acid for some strains of H. pylori (41). These ligands have been shown to be present in the stomach during infection via metabolomics studies (48, 49); however, the exact concentration of these ligands is not known. Each chemotaxis-active TlpA ligand is important for critical cellular functions for H. pylori; therefore, the ability to sense these ligands is a likely survival-linked evolutionary adaptation.

### TlpA appears to use both binding pockets.

dCache_1 chemoreceptors contain two potential ligand binding pockets in each Cache subdomain. Previous work showed that dCache_1 chemoreceptors sense chemotaxis-active ligands through either subdomain but, as of yet, not both (20, 21). In contrast, our data suggest that TlpA might bind ligands in both Cache subdomains. Ligand binding locations, suggested by docking prediction analysis, placed fumarate within the membrane-proximal subdomain and arginine within the membrane-distal subdomain. These location assignments were further supported by TlpA_LBD_ point mutants of residues within the predicted dCache_1 membrane-distal (D165A and M183A) and membrane-proximal (Y228A, Y252A, and D254A) subdomains. All membrane-distal subdomain mutations led to a decreased binding affinity for the chemotaxis-active ligands arginine, fumarate, and cysteine as well as the antagonist ligand glucosamine. These findings suggest that the membrane-distal site is important for chemotaxis signaling, as seen in other dCache_1 receptors (18, 21). It has been shown that individual dCache_1 receptors can bind aliphatic, small polar, and large positively charged amino acids through a single subdomain due to the malleable nature of dCache_1 receptors that can accommodate ligands of different sizes and charges (40). Thus, it is plausible that the membrane-distal subdomain could be able to accommodate these diverse ligands.

Our data also suggest that the membrane-proximal subdomain plays a role in TlpA ligand binding. Site-directed mutagenesis of the membrane-proximal subdomain, as well as the membrane-distal one, decreased fumarate binding. This outcome suggests that the point mutations either directly disrupted ligand binding or altered long-range interactions in the protein that influence ligand binding affinities. Additionally, our data showed that fumarate blocked arginine binding despite having similar binding affinities. Of note, arginine binding to the membrane-proximal domain was affected by the membrane-proximal domain point mutant TlpA_Y228A_, while fumarate was affected by both the TlpA_Y228A_ and TlpA_D254A_ membrane-proximal domain point mutants, which supports docking analysis predictions suggesting that fumarate preferentially binds to the membrane-proximal domain, while arginine preferentially binds to the membrane-distal domain. Several models could account for these findings. One is that fumarate binds at the membrane-proximal site and creates an allosteric change that prevents ligand binding at the membrane-distal site. Alternatively, the amino acid changes in the proximal site could affect the ligand affinities at the distal site. Finally, a third possibility is that arginine binds at both sites, and proximal-site binding is affected by the proximal mutations. It will be interesting to dissect whether there are cooperative interactions, distinct site binding, or simultaneous site binding. Regardless, while cooperativity between subdomains in dCache_1 chemoreceptors has not yet been observed, it is well documented that four-helix-bundle types of chemoreceptors have negative cooperativity between their two binding sites (34). Furthermore, it is not yet known which site is required for chemotaxis. Overall, our data suggest that TlpA may use both dCache_1 subdomains to bind ligands.

### TlpA chemotaxis responses can be antagonized.

We were somewhat surprised to find a high-affinity-binding TlpA ligand, glucosamine, that did not elicit a chemotaxis response yet appeared to bind the membrane-distal dCache_1 subdomain. Indeed, we found that glucosamine occluded chemotaxis-active TlpA ligands from binding and inhibited the normal chemoattractant responses to arginine, fumarate, and cysteine. This response was observed regardless of whether glucosamine was added before or after the addition of the TlpA chemoattractants, suggesting that glucosamine may have a very high on-rate for binding TlpA compared to arginine, fumarate, or cysteine. Two studies have reported high-affinity chemoreceptor ligands that acted as antagonists by blocking chemotaxis-active ligand binding (37, 38), and Cache receptor antagonists have been reported for a histidine kinase (50). Martín-Mora and colleagues showed that the binding of the attractant malic acid to the P. aeruginosa sCache chemoreceptor PA2652 was inhibited by either citraconic acid or d,l-methylsuccinic acid, and subsequently, chemoattractant responses to malic acid were decreased (37). Another of these studies described finding diverse ligands for the E. coli four-helix-bundle chemoreceptor Tar and reported that a high-affinity ligand, *cis*-1,2-cyclohexane-dicarboxylic acid, also acted as an antagonist for aspartate chemotaxis. *cis*-1,2-Cyclohexane-dicarboxylic acid competed for aspartate binding and blocked intracellular kinase activity (38). We expect that there will be more discoveries of these types of chemomodulatory antagonists, or maybe even chemotaxis-enhancing ligands, because ligand discovery methods have changed. Specifically, previous efforts relied on chemotaxis assays, and so only chemotaxis-active ligands could be identified. In contrast, recent approaches look for direct ligand-receptor interactions at the molecular level, thus expanding our ability to identify the interacting partners (21, 38, 51–53).

The function of chemoreceptor antagonists is not yet known in any system (37, 38). In the case of TlpA, it is possible to speculate that when confronted with abundant glucosamine, H. pylori benefits by not responding to arginine, fumarate, or cysteine. However, the role of glucosamine in H. pylori infection is unknown, although it has been shown to support the growth of some H. pylori clinical isolates using phenotypic Biolog plates (54). One possibility is that antagonist ligands may function as a form of adaptation, as H. pylori lacks the classical adaptation proteins CheR and CheB (55), possibly in lieu of, or in augmentation to, other adaptation systems. Chemotaxis antagonists may be useful tools to modulate chemotaxis and affect bacterial pathogenesis. In the case of TlpA, blocking its function early in infection would decrease colonization; however, later attenuation of chemotactic responses might be predicted to enhance inflammation (15–17). Future work performing molecular dynamics experiments to understand how agonist and antagonist ligands interact with TlpA_LBD_ will help us to understand the function of chemoreceptor antagonists.

One caveat of this study is that experimental analyses were carried out under two different conditions. The ligand binding work was all done with purified TlpA_LBD_, while the chemotaxis studies were done on full-length TlpA that was in the context of both a membrane and its interactions with other chemoreceptors. These two different situations may lead to varying outcomes. For example, it is not clear how ligand interactions would change in the context of chemosensory array-packed receptors, which is an area for future work.

Expanding the knowledge of TlpA ligands, and how TlpA interacts with these ligands, is essential for better understanding why TlpA enhances the *in vivo* fitness of H. pylori and alters inflammatory phenotypes driven by H. pylori. Future experiments manipulating the ability of H. pylori to sense specific TlpA ligands will be useful to understand whether all or a subset of TlpA ligands play a role in driving these *in vivo* phenotypes (15–17). Furthermore, this work provides another example (37, 38) of a chemotaxis system having antagonistic ligands, operating through a distinct type of chemoreceptor ligand binding domain. These results suggest an interesting possible mechanism for regulating responses to multiple chemotactic ligands in a nutrient-rich environment using agonist and antagonist ligands.

## MATERIALS AND METHODS

### TlpA construct design and protein purification.

The periplasmic portion of TlpA (TlpA_LBD_), amino acids 28 to 299, from Helicobacter pylori SS1 was cloned into a pBH4 expression vector (pBH4_TlpA_LBD_) to generate an N-terminal 6×His-tagged construct, with a tobacco etch virus (TEV) protease site, under the control of isopropyl-β-d-thiogalactopyranoside (IPTG) (19). Alanine point mutants at Asp165, Met183, Tyr228, Tyr252, and Tyr254 in TlpA_LBD_ were generated via site-directed mutagenesis of pBH4_TlpA_LBD_ with primers listed in Table S4 in the supplemental material and confirmed via restriction digestion and sequencing (QuikChange; Stratagene). TlpA_LBD_ and all point mutants were purified as described previously by Sweeney et al. (19). Circular dichroism (CD) spectroscopy was used to confirm the correct folding of all proteins (Fig. S3) (56).

10.1128/mBio.01819-21.3 FIG S3 CD spectrometry of WT TlpA_LBD_ and point mutants TlpA_D165A_ and TlpA_Y252A_. The far-UV CD spectra of TlpA_D165A_ and TlpA_Y252A_ are similar to WT TlpA_LBD_, including all other TlpA_LBD_ point mutants used in this study (data not shown). Download FIG S3, PDF file, 0.6 MB. Copyright © 2021 Johnson et al. 2021 Johnson et al. https://creativecommons.org/licenses/by/4.0/ This content is distributed under the terms of the Creative Commons Attribution 4.0 International license.

10.1128/mBio.01819-21.8 TABLE S4 Primers for site-directed mutagenesis of TlpA_LBD_. Mutated codons, bolded; restriction sites used for screening clones, underlined. Download Table S4, PDF file, 0.4 MB. Copyright © 2021 Johnson et al. 2021 Johnson et al. https://creativecommons.org/licenses/by/4.0/ This content is distributed under the terms of the Creative Commons Attribution 4.0 International license.

### Ligand binding array.

Small-molecule arrays were prepared and performed as previously described (57). Briefly, 1 μg of purified TlpA_LBD_ in phosphate-buffered saline (PBS) (pH 7.2) was incubated with a molar concentration ratio (4:2:1) of anti-His antibody (Cell Signaling), followed by incubation with a secondary antibody (Thermo Scientific) for signal amplification. The protein-antibody mix was added to an Arrayit Superepoxy III glass substrate array blocked with PBS (pH 7.2) with 1% bovine serum albumin. The glass substrate array was printed with quadruplicate spots of 148 different amino acids, salts of organic acids, and other small molecules (Table S1). Unbound protein was washed away with PBS with 0.05% Tween. The arrays were scanned by a ProScan array scanner at 488/520 nm, and the results were analyzed by the ScanArray Express software program (PerkinElmer). Three biological replicates were performed, with a total of 12 data points for each glycan tested. Binding was classified as positive for a ligand if the relative fluorescence unit value was >1-fold above the mean background (defined as the average background of negative-control spots plus 3 standard deviations [SD]) and was statistically significant (*P < *0.005 by Student’s *t* test). Small-molecule array slide preparation and analysis were done according to MIRAGE guidelines (58) (outlined in Table S5).

10.1128/mBio.01819-21.9 TABLE S5 Supplementary glycan microarray document based on MIRAGE guidelines (Y. Liu, R. McBride, M. Stoll, A. S. Palma, et al., Glycobiology 27:280–284, 2017, https://doi.org/10.1093/glycob/cww118). Download Table S5, PDF file, 0.8 MB. Copyright © 2021 Johnson et al. 2021 Johnson et al. https://creativecommons.org/licenses/by/4.0/ This content is distributed under the terms of the Creative Commons Attribution 4.0 International license.

### Surface plasmon resonance measurements.

Purified TlpA_LBD_ was immobilized on a CM5 series S sensor chip, and binding affinities were tested using a Biacore S200 instrument (GE Healthcare) as described previously (39, 57, 59). Briefly, proteins were captured using an amine-coupling kit (GE Healthcare), in which the carboxylmethyl dextran matrix of the sensor chip was activated by the injection of a mixture of 0.2 M 1-ethyl-3-[(3-dimethylamino)propyl]-carbodiimide (EDC) and 0.05 M *N*-hydroxysuccinimide (NHS), followed by the neutralization of the remaining unreacted NHS ester groups by the injection of 1 M ethanolamine-HCl (pH 8.0). Purified TlpA_LBD_ was diluted in 10 mM sodium acetate buffer (pH 4.5) at a concentration of 100 μg/ml for immobilization to the chip. A total of 8,400 response units (RU) of TlpA_LBD_ were captured on flow cell 2. As a negative control, flow cell 1 was a blank control undergoing the same treatment as the other flow paths, without the protein injection. This set enabled double-reference subtraction of the responses (2 − 1, 3 − 1, and 4 − 1). The tested compounds were prepared as a stock concentration of 100 to 200 mM in PBS. The compounds were then diluted between 1 nM and 1 mM in a series of 1:10 dilutions in PBS and run over the flow cells at a flow rate of 30 μl/min. Between each sample testing, a series of buffer-only injections was run to enable double-blank subtraction for the sensorgram assessment. After the initial run, based on the results, the dilution series ranged from 0.195 μM to 1 mM in 1:4 dilutions in PBS. The samples were then run using single-cycle kinetic/affinity methods in triplicate for those compounds that showed submillimolar affinity after the initial binding screen. The data sets were analyzed using Biacore S200 evaluation software 2.0.2; sensorgrams were double-reference subtracted.

### Bacterial strains and growth conditions.

For all chemotaxis assays, H. pylori strain PMSS1 was used (9). Bacteria were grown in Brucella broth (BD BBL/Fisher) with 10% heat-inactivated fetal bovine serum (FBS) (Life Technologies) (BB10), with shaking, at 37°C under microaerobic conditions of 5% O_2_, 10% CO_2_, and 85% N_2_. The PMSS1 Δ*tlpA* mutant was created by the natural transformation of wild-type PMSS1 with 5 μg of Δ*tlpA*::*cat* SS1 genomic DNA (16). Chloramphenicol-resistant mutants were selected using 10 μg/ml chloramphenicol on Columbia horse blood agar as previously described (16). Mutation of *tlpA* was confirmed by PCR amplification of genomic DNA from WT PMSS1, Δ*tlpA*::*cat* PMSS1, and Δ*tlpA*::*cat* SS1 using primers TlpA_SS1_5′ (TTGTCTAAAGGTTTGAGTATC) and TlpA_SS1_3′ (TTAAAACTGCTTTTTATTCAC) (this study) (Fig. S4).

10.1128/mBio.01819-21.4 FIG S4 Verification of Δ*tlpA*::*cat* PMSS1. Mutation of *tlpA* was confirmed by PCR amplification of gDNA from WT PMSS1, Δ*tlpA*::*cat* PMSS1, and Δ*tlpA*::*cat* SS1 (T. M. Andermann, Y.-T. Chen, and K. M. Ottemann, Infect Immun 70:5877–5881, 2002, https://doi.org/10.1128/IAI.70.10.5877-5881.2002) using primers TlpA_SS1_5′ (TTGTCTAAAGGTTTGAGTATC) and TlpA_SS1_3′ (TTAAAACTGCTTTTTATTCAC). Download FIG S4, PDF file, 0.6 MB. Copyright © 2021 Johnson et al. 2021 Johnson et al. https://creativecommons.org/licenses/by/4.0/ This content is distributed under the terms of the Creative Commons Attribution 4.0 International license.

### Chemotaxis assays.

Swimming behavior assays were done with H. pylori PMSS1 strains grown in BB10 as described above. Cultures grown overnight were diluted to an optical density at 600 nm (OD_600_) of 0.1 in fresh BB10 and then incubated with shaking as described above until an OD_600_ of 0.12 to 0.15 was reached. The motility of these cultures was confirmed, and they were then used for chemotaxis assays by treating them with l-arginine monohydrochloride (catalog number B577-05; J. T. Baker), sodium fumarate (catalog number 215531000; Acros Organics), l-cysteine hydrochloride monohydrate (catalog number C81020; RPI), d(+)-glucosamine hydrochloride (catalog number 01450; Chem-Impex International Inc.), thiamine hydrochloride (catalog number BP892; Fisher BioReagents), α-ketoglutaric acid (catalog number SC-208504; Santa Cruz Biotechnology), or l-malic acid (catalog number 102237; MP Biomedicals) at a final concentration of 0.1 mM, 1 mM, or 10 mM or with an equal volume of H_2_O as a mock-treated control (4 μl H_2_O or 4 μl of a ligand stock in H_2_O into a 96-μl culture). The number of direction changes in a bacterial swimming trajectory was enumerated over a 3-s interval to determine whether each putative ligand is sensed as an attractant or repellent or elicits no response (20, 25–30). The results were compared to those with both a repellent control, 10 mM HCl (catalog number A144S; Fisher Chemical), which results in increased direction changes (29, 31), and an attractant control, 50 μM 2,2′-dipyridyl (catalog number 117500250; Arcos Organics), which results in fewer direction changes (25). Each control is sensed by chemoreceptors other than TlpA (24, 25, 29, 31). The pH of BB10 upon treatment was independently assessed using a Denver Instruments pH meter. Prior to realizing that chemotactic responses may be due to medium acidification, we resuspended all ligands in pure water. Therefore, to be able to compare the results to those of previous experiments, we continued resuspending acidified ligands in pure water and then adjusting the pH of the resuspended ligand using NaOH. Cultures were filmed immediately after ligand addition at a ×400 magnification using a Hamamatsu C4742-95 digital camera with μManager software (version 1.4.22), mounted on a Nikon Eclipse E600 phase-contrast microscope. For the competition chemotaxis assay, cultures of H. pylori and ligands were prepared as described above. However, 1 min after the addition of a nonchemoactive ligand at a final concentration of 10 mM, a chemoactive ligand was added at a final concentration of 10 mM, and the cultures were then filmed as described above. Videos were relabeled to blind the observer to the strain identity. For each sample, >100 3-s-long bacterial tracks from three independent cultures were analyzed manually to identify stops followed by direction changes. Data for all biological replicates under each condition were combined, and the average number of direction changes in 3 s and the standard error of the mean were calculated. For each strain, data were normalized to the values for the untreated control under each experimental condition. Statistical analysis of the data for treated versus untreated samples was performed using two-way analysis of variance (ANOVA) and Dunnett’s multiple-comparison test.

### SPR TlpA competition assays.

SPR competition assays were performed by using a Biacore S200 instrument and the A-B-A inject function (56). Competition A-B-A analyses were used to interrogate the specificity of the potential ligand binding site preferences of TlpA_LBD_ and to unravel the nature of the ligand-sensor interactions. This assay was designed to show if a cumulative response is observed when a second analyte (B) is flown across the bound protein saturated with the first analyte (A) (Fig. 4). As the assay is designed to provide saturation of all analytes tested, this assay does not provide 1:1 competition to indicate which is the preferred analyte for a binding site. The wild-type TlpA_LBD_ protein was immobilized as described above. A-B-A was used with combinations of each of the compounds (at a concentration 10-fold higher than the equilibrium dissociation constant [*K_D_*]) and the PBS control, with 60-s injections of analyte A to ensure that saturation or near saturation was reached prior to competition with analyte B. The results were analyzed using Biacore S200 evaluation software in the sensorgram mode, and data were zeroed to the baseline before the initial analyte A injection. All response data were normalized to a molecular weight of 100 Da for each analyte, allowing direct comparison of responses. Independent-site theoretical values are calculated by taking the sum of individual responses. Shared-site theoretical values are calculated by taking the sum of individual responses divided by the number of individual responses.

### Saturation transfer difference NMR.

In the saturation transfer difference (STD) NMR experiment, the entire TlpA_LBD_ protein was first saturated at the protein resonances, and excess ligand was then added. As the ligand binds and releases from the receptor, saturation transfers from the protein to the bound ligand. This transfer appeared as an increase in the ligand intensity on epitopes that interacted with the TlpA_LBD_ protein. For STD NMR experiments, samples of 25 μM TlpA_LBD_ in complex with either 2.5 mM arginine (Arg) or fumaric acid (Fum) in 99% D_2_O were prepared. All STD NMR spectra were acquired in Shigemi tubes (Shigemi, USA) with a Bruker 600-MHz Advance spectrometer at 283 K using a 1^H^-13^C^-15^N^ gradient cryoprobe equipped with z-gradients. Protein resonances were saturated at −1.0 ppm (on-resonance) and 33 ppm (off-resonance), with a total saturation time of 2 s. A total of 512 scans per STD NMR experiment were acquired, and a Watergate sequence was used to suppress the residual HDO signal. A spin-lock filter with a 5-kHz strength and a duration of 10 ms was applied to suppress the protein background. On- and off-resonance spectra were stored and processed separately, and the final STD NMR spectra were obtained by subtracting the on- and off-resonance spectra. Control STD NMR experiments were performed identically in the absence of protein.

### Docking analysis.

To evaluate a potential binding site for Arg and Fum with TlpA_LBD_ (PDB accession number 6E09), a blind docking experiment was performed using the AutoDock Vina protocol (60), a high-scoring molecular docking program (32), implemented in the YASARA structure molecular modeling package (version 16.46) (33). The blind docking experiment was set up by using the entire TlpA protein as a potential binding site (grid size, 92.99 Å by 75.73 Å by 62.13 Å). A total of 999 Vina docking runs were performed.
