# Supplementary figures and images for: The dCache Chemoreceptor TlpA of Helicobacter pylori Binds Multiple Attractant and Antagonistic Ligands via Distinct Sites
Source: mBio. 2021 Aug 3;12(4):e01819-21. doi: 10.1128/mBio.01819-21 (PMC8406319; doi:10.1128/mBio.01819-21)

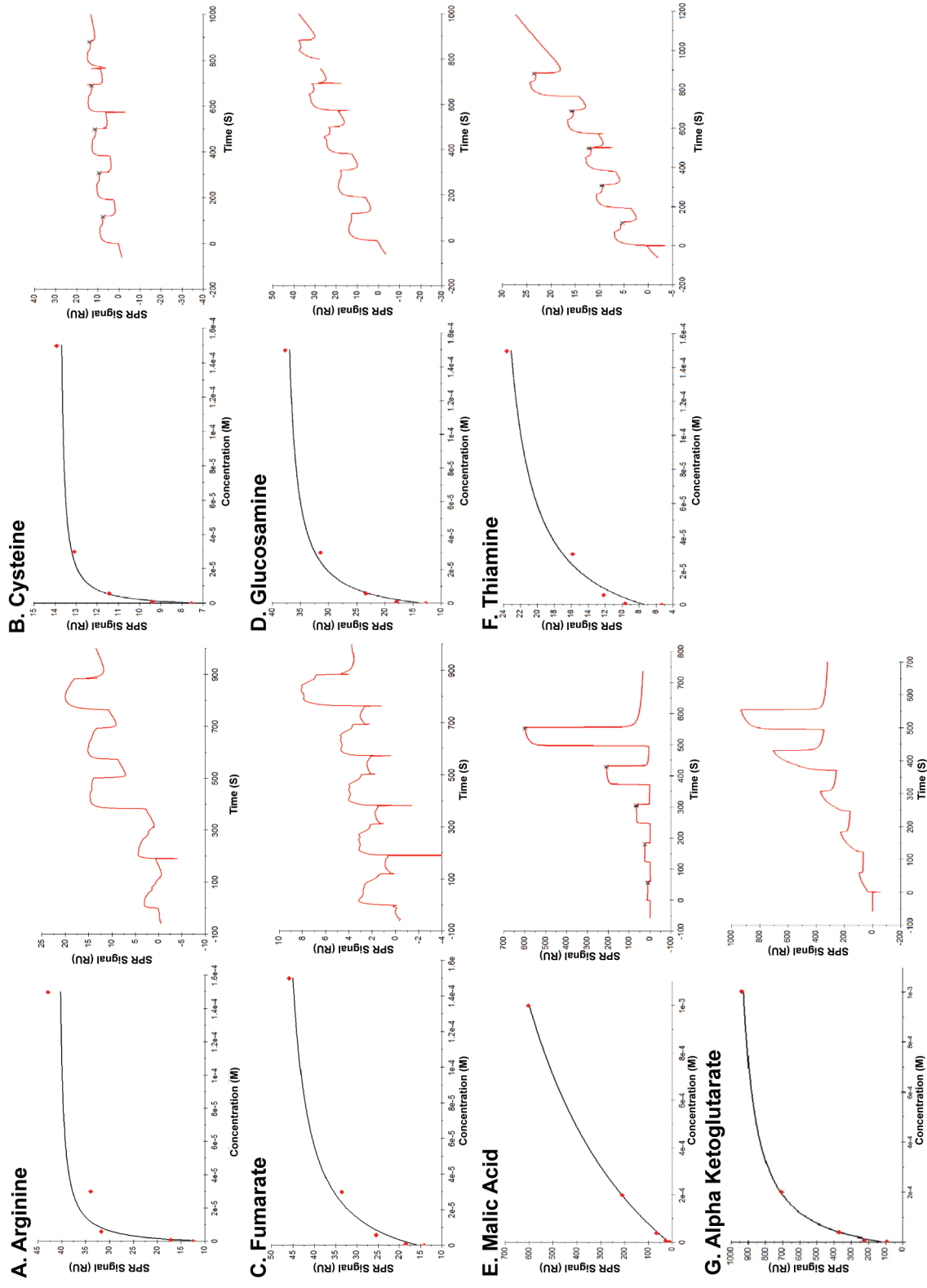

Supplement: FIG S1 [file mbio.01819-21-sf001.pdf]

**A.**

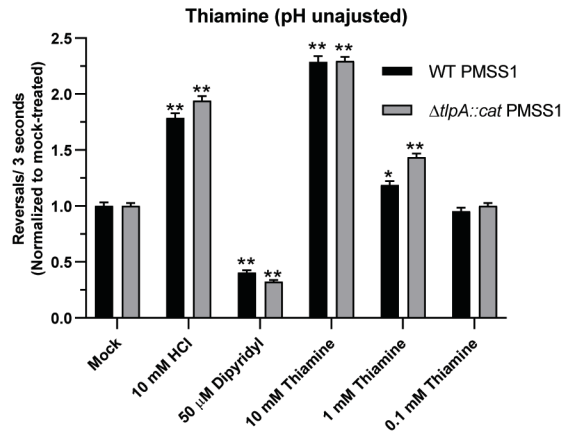

**B.**

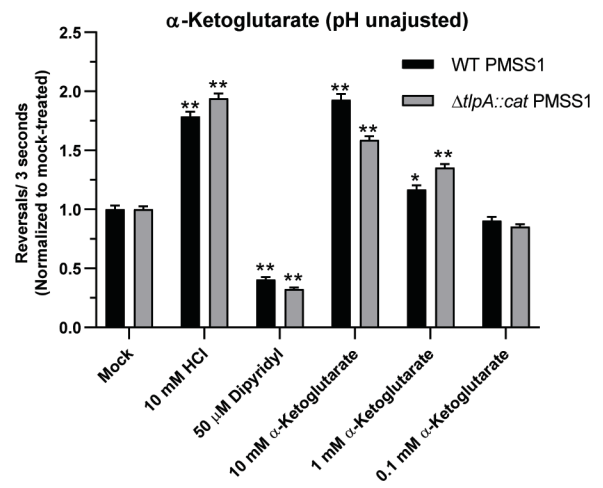

**C.**

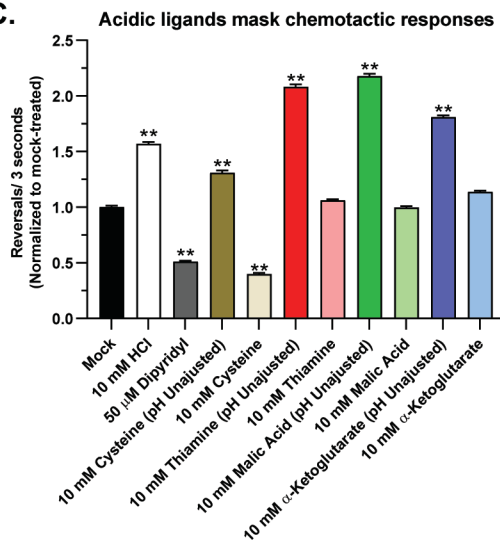

Supplement: FIG S2 [file mbio.01819-21-sf002.pdf]

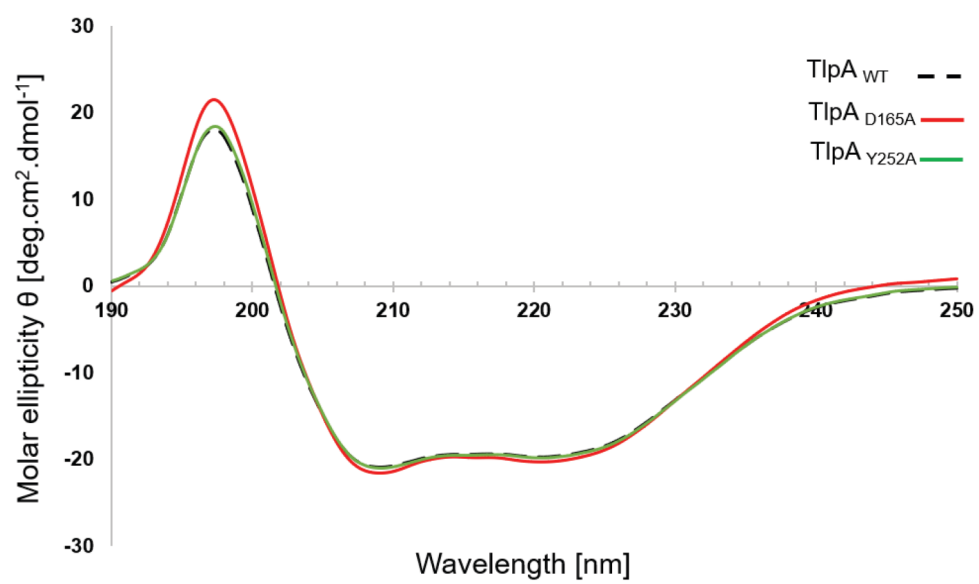

Supplement: FIG S3 [file mbio.01819-21-sf003.pdf]

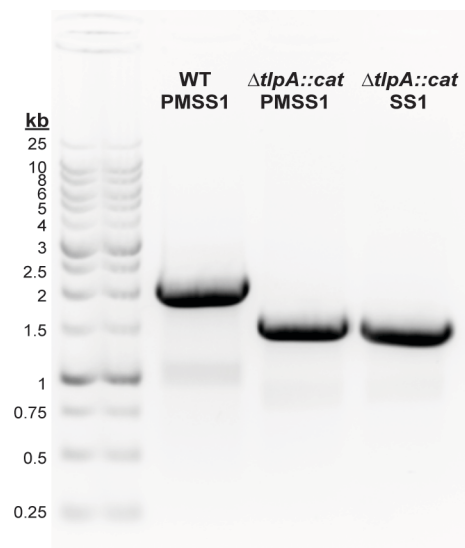

Supplement: FIG S4 [file mbio.01819-21-sf004.pdf]
