# Supplementary material for: The dCache Chemoreceptor TlpA of Helicobacter pylori Binds Multiple Attractant and Antagonistic Ligands via Distinct Sites
Source: mBio. 2021 Aug 3;12(4):e01819-21. doi: 10.1128/mBio.01819-21 (PMC8406319; doi:10.1128/mBio.01819-21)
Supplement: TABLE S1 [file mbio.01819-21-st001.pdf]

Name	Structure/ Molecular Formula
**Amino acids, salts of organic acids, + other small molecules**	
Alanine	$C_3H_7NO_2$
Arginine	$C_6H_{14}N_4O_2$
Asparagine	$C_4H_8N_2O_3$
Aspartate	$C_4H_7NO_4$
Cysteine	$C_3H_7NO_2S$
Fumaric Acid	$C_4H_4O_4$
Glucosamine	$C_6H_{13}NO_5$
Glutamic acid	$C_5H_9NO_4$
Glutamine	$C_5H_{10}N_2O_3$
Histidine	$C_6H_9N_3O_2$
Isoleucine	$C_6H_{13}NO_2$
leucine	$C_6H_{13}NO_2$
Lysine	$C_6H_{14}N_2O_2$
Malic Acid	$C_4H_6O_5$
Methionine	$C_5H_{11}NO_2S$
Phenylalanine	$C_9H_{11}NO_2$
Proline	$C_5H_9NO_2$
Purine	$C_5H_4N_4$
Serine	$C_3H_7NO_3$
Succinic Acid	$C_4H_6O_4$
Thiamine	$C_{12}H_{17}N_4OS$
Threonine	$C_4H_9NO_3$
Tryptophan	$C_{11}H_{12}N_2O_2$
Tyrosine	$C_9H_{11}NO_3$
Valine	$C_5H_{11}NO_2$
α-ketoglutarate	$C_5H_6O_5$
**Terminal Galactose**	
Lacto-*N*-Biose I	Galβ1-3Glc*N*Ac
N-Acetyllactosamine	Galβ1-4Glc*N*Ac
β1-4galactosyl-galactose	Galβ1-4Gal
β1-6galactosyl-*N*-acetylglucosamine	Galβ1-6Glc*N*Ac
β1-3galactosyl-*N*-acetylgalactosamine	Galβ1-3Gal*N*Ac
AsialoG_{M1}	Galβ1-3Gal*N*Acβ1-4Galβ1-4Glc
Lacto-*N*-tetrose	Galβ1-3Glc*N*Acβ1-3Galβ1-4Glc
Lacto-*N*-neotetrose	Galβ1-4Glc*N*Acβ1-3Galβ1-4Glc
Lacto-*N*-neohexose	Galβ1-4Glc*N*Acβ1-6(Galβ1-4Glc*N*Acβ1-3)Galβ1-4Glc
Lacto-*N*-hexose	Galβ1-4Glc*N*Acβ1-6(Galβ1-3Glc*N*Acβ1-3)Galβ1-4Glc
Globotriose	Gala 1-4Galβ1-4Glc

Tn Antigen	Gal*N*Aca1-*O*-Ser
Galactosyl-Tn Antigen	Galb1-3Gal*N*Acα1-*O*-Ser
α1-3 Galactobiose	Gala1-3Gal
Linear B-2 Trisaccharide	Gala1-3Galβ1-4Glc*N*Ac
Linear B-6 Trisaccharide	Gala1-3Galβ1-4Glc
α1-3, β1-4, α1-3 Galactotetrose	Gala1-3Galβ1-4Galα1-3Gal
β1-6Galactobiose	Galβ1-6Gal
Terminal disaccharide of globotriose	Gal*N*Acβ1-3Gal
Receptor for pili of *P. aeruginosa*	Gal*N*Acβ1-4Gal
P1 Antigen	Galα1-4Galβ1-4Glc*N*Ac
α-D-*N*-acetylgalactosaminyl-1-3Gal-β1-4Glc	Gal*N*Acα1-3Galβ1-4Glc
iso-Lacto-*N*-octose	Galβ1-3Glc*N*Acβ1-3Galβ1-4Glc*N*Acβ1-6(Galβ1-3Glc*N*Acβ1-3)Galβ1-4Glc
para-Lacto-*N*-hexose	Galβ1-3Glc*N*Acβ1-3Galβ1-4Glc*N*Acβ1-3Galβ1-4Glc
**Terminal *N*`Acetyl glucosamine**	
N,N′-Diacetyl chitobiose	Glc*N*Acβ1-4Glc*N*Ac
N,N′,N″-Triacetyl chitotriose	Glc*N*Acβ1-4Glc*N*Acβ1-4Glc*N*Ac
N,N′,N″,N‴-Tetraacetyl chitotetrose	Glc*N*Acβ1-4Glc*N*Acβ1-4Glc*N*Acβ1-4Glc*N*Ac
N,N′,N″,N‴,N″″,N″‴-Hexaacetyl chitohexose	Glc*N*Acβ1-4Glc*N*Acβ1-4Glc*N*Acβ1-4Glc*N*Acβ1-4Glc*N*Acβ1-4Glc*N*Ac
Bacterial cell wall muramyl discaccharide	Glc*N*Acβ1-4Mur*N*Ac
**Mannose containing structures**	
β1-2-*N*-Acetylglucosamine-mannose	Glc*N*Acβ1-2Man
Bianntennary *N*-linked core pentasaccharide	Glc*N*Acβ1-2Manα1-6(Glc*N*Acβ1-2Manα1-3)Man
α1-2-Mannobiose	Manα1-2Man
α1-3-Mannobiose	Manα1-3Man
α1-4-Mannobiose	Manα1-4Man
α1-6-Mannobiose	Manα1-6Man
α1-3, a1-6-Mannobiose	Manα1-6(Manα1-3)Man
α1-3, α1-3, α1-6-Mannopentaose	Manα1-6(Manα1-3)Manα1-6(Manα1-3)Man
**Fucosylated structures**	
Lacto-*N*-fucopentose I	Fucα1-2Galβ1-3Glc*N*Acβ1-3Galβ1-4Glc
Lacto-*N*-fucopentose II	Galβ1-3(Fuca1-4)Glc*N*Acβ1-3Galβ1-4Glc
Lacto-*N*-fucopentose III	Galβ1-4(Fuca1-3)Glc*N*Acβ1-3Galβ1-4Glc
Lacto-*N*-difucohexose I	Fucα1-2Galβ1-3(Fucα1-4)Glc*N*Acβ1-3Galβ1-4Glc
Lacto-*N*-difucohexose II	Galβ1-3(Fucα1-4)Glc*N*Acβ1-3Galβ1-4(Fucα1-3)Glc
H-disaccharide	Fucα1-2Gal
2'-Fucosyllactose	Fucα1-2Galβ1-4Glc
3'-Fucosyllactose	Galβ1-4(Fucα1-3)Glc
Lewis^{x}	Galβ1-4(Fucα1-3)Glc*N*Ac
Lewis^{a}	Galβ1-3(Fucα1-4)Glc*N*Ac

Blood Group A-trisaccharide	Gal*N*Acα1-3(Fucα1-2)Gal
Lactodifucotetrose	Fucα1-2Galβ1-4(Fucα1-3)Glc
Blood Group B-Trisaccharide	Galβ1-3(Fucα1-2)Gal
Lewis[y]	Fucα1-2Galβ1-4(Fucα1-3)Glc*N*Ac
Blood Group H Type II Trisaccharide	Fucα1-2Galβ1-3Glc*N*Ac
Lewisb tetrasaccharide	Fucα1-2Galβ1-3(Fucα1-4)Glc*N*Ac
Sulpho Lewis[a]	SO_3-3Galβ1-3(Fucα1-4)Glc*N*Ac
Sulpho Lewis[x]	SO_3-3Galβ1-4(Fucα1-3)Glc*N*Ac
Monofucosyl-para-Lacto-*N*-hexose IV	Galβ1-3Glc*N*Acβ1-3Galβ1-4(Fucα1-3)Glc*N*Acβ1-3Galβ1-4Glc
Monofucosyllacto-*N*-hexose III	Galβ1-4(Fucα1-3)Glc*N*Acβ1-6(Galβ1-3Glc*N*Acβ1-3)Galβ1-4Glc
Difucosyllacto-*N*-hexose	Galβ1-4(Fucα1-3)Glc*N*Acβ1-6(Fucα1-2Galβ1-3Glc*N*Acβ1-3)Galβ1-4Glc
Trifucosyllacto-*N*-hexose	Galβ1-4(Fucα1-3)Glc*N*Acβ1-6(Fucα1-2Galβ1-3(Fucα1-4)Glc*N*Acβ1-3)Galβ1-4Glc
Lacto-*N*-fucopentaose VI	Galβ1-4GlcNAcβ1-3Galβ1-4(Fucα1-3)Glc
Lacto-*N*-neodifucohexaose I	Fucα1-2Galβ1-4(Fucα1-3)GlcNAcβ1-3Galβ1-4Glc
Lacto-*N*-neodifucohexaose II	Fucα1-3Galβ1-4GlcNAcb1-3Galβ1-4(Fucα1-3)Glc
Trifucosyllacto-*N*-neoteraose I	Fucα1-2Galβ1-4(Fucα1-3)GlcNAcβ1-3(Fucα1-2)Galβ1-4Glc
Monofucosyllacto-*N*-neohexaose I	Galβ1-4(Fucα1-3)GlcNAcβ1-6(Galβ1-4GlcNAcβ1-3)Galβ1-4Glc
Difucosyllacto-*N*-neohexaose I	Galβ1-4(Fucα1-3)GlcNAcβ1-6(Galβ1-4(Fucα1-3)GlcNAcβ1-3)Galβ1-4Glc
Difucosyllacto-*N*-neohexaose II	Fucα1-2Galβ1-4(Fucα1-3)GlcNAcβ1-6(Galβ1-4GlcNAcβ1-3)Galβ1-4Glc
Monofucosyl(1-3)-iso-lacto-*N*-octaose	Galβ1-3GlcNAcβ1-3Galβ1-4(Fucα1-3)GlcNAcβ1-6(Galβ1-3GlcNAcβ1-3)Galβ1-4Glc
Trifucosyl(1-2,1-2,1-3)-iso-lacto-N-octaose	Fucα1-2Galβ1-3GlcNAcβ1-3Galβ1-4(Fucα1-3)GlcNAcβ1-6(Galβ1-3GlcNAcβ1-3)Galβ1-4Glc
Blood Group A Tetrasaccharide	GalNAcβ1-3(Fucα1-2)Galβ1-4Glc
Blood Group B pentasaccharide	Galβ1-3(Fucα1-2)Galβ1-4(Fucα1-3)Glc
**Neu5Ac containing structures**	
Sialyl Lewis[a]	Neu5Acα2-3Galβ1-3(Fucα1-4)Glc*N*Ac
Sialyl Lewis[x]	Neu5Acα2-3Galβ1-4(Fucα1-3)Glc*N*Ac
Sialyllacto-*N*-tetrose a	Neu5Acα2-3Galβ1-3Glc*N*Acβ1-3Galβ1-4Glc
Monosialyl, monofucosyllacto-*N*-neohexose	Galβ1-4(Fucα1-3)Glc*N*Acβ1-6(Neu5Acα2-6Galβ1-4Glc*N*Acβ1-3)Galβ1-4Glc
2,3'-Sialyllactosamine	Neu5Acα2-3Galβ1-4Glc*N*Ac
2,6'-Sialyllactosamine	Neu5Acα2-6Galβ1-4Glc*N*Ac
LS-Tetrasaccharide a	Neu5Acα2-3Galβ1-3GlcNAcβ1-3Galβ1-4Glc

LS-Tetrasaccharide b	Galβ1-3(Neu5Acα2-6)Glc*N*Acβ1-3Galβ1-4Glc
LS-Tetrasaccharide c	Neu5Acα2-6Galβ1-4Glc*N*Acβ1-3Galβ1-4Glc
Disialyllacto-*N*-tetrose	Neu5Acα2-3Galβ1-3(Neu5Acα2-6)Glc*N*Acβ1-3Galβ1-4Glc
2,3'-Sialyllactose	Neu5Acα2-3Galβ1-4Glc
2,6'-Sialyllactose	Neu5Acα2-6Galβ1-4Glc
Colominic acid	(Neu5Acα2-8Neu5Ac)n (n<50)
Biantennary 2,6-sialylated-*N*-glycan-Asn	Neu5Acα2-6Galβ1-4Glc*N*Acβ1-2Manα1-6(Neu5Acα2-6Galβ1-4Glc*N*Acβ1-2Manα1-6)Manβ1-4Glc*N*Acβ1-4Glc*N*Ac-Asn
**Carageenan and Glycoaminoglycans (GAGS)**	
Neocarratetrose-41, 3-di-*O*-sulphate (Na^+)	$C_{24}H_{36}O_{25}S_2Na_2$ (Mixed anomers. Tetrasaccharide of regular κ - carrageenan)
Neocarratetrose-41-*O*-sulphate (Na^+)	$C_{24}H_{37}O_{22}SNa$ (Mixed anomers. Derived from C1003 by removal of the non-reducing terminal 4-sulphate)
Neocarrahexose-24,41, 3, 5-tetra-*O*-sulphate (Na^+)	$C_{36}H_{52}O_{40}S_4Na_4$ (Mixed anomers. A hybrid sequence comprising carrageenan disaccharides in the order k-i-k, derived from the carrageenan from *Chondrus crispus*)
Neocarrahexose-41, 3, 5-tri-*O*-sulphate (Na^+)	$C_{36}H_{53}O_{37}S_3Na_3$ (Mixed anomers. Hexasaccharide of regular κ-carrageenan)
Neocarraoctose-41, 3, 5, 7-tetra-*O*-sulphate (Na^+)	$C_{48}H_{70}O_{49}S_4Na_4$ (Mixed anomers. Octasaccharide of regular κ-carrageenan)
Neocarradecose-41, 3, 5, 7, 9-penta-*O*-sulphate (Na^+)	$C_{60}H_{87}O_{61}S_5Na_5$ (Mixed anomers. Decasaccharide of regular κ- carrageenan)
ΔUA-2S → Glc*N*S-6S Na_4 (I-S)	$C_{12}H_{15}NO_{19}S_3Na_4$ (Predominant disaccharide produced from heparin by heparinase I and II)
ΔUA → Gluc*N*S-6S Na_3 (II-S)	$C_{12}H_{16}NO_{16}S_2Na_3$ (Produced from heparinase II digestion of heparin and heparin sulphate)
ΔUA → 2S-Glc*N*S Na_3 (III-S)	$C_{12}H_{16}NO_{16}S_2Na_3$ (Produced from heparin by digestion with heparinase I and II)
ΔUA → 2S-Glc*N*Ac-6S Na_3 (I-A)	$C_{14}H_{18}NO_{17}S_2Na_3$ (Minor component produced from heparin by heparinase II)
ΔUA → Glc*N*Ac-6S Na_2 (II-A)	$C_{14}H_{19}NO_{14}SNa_2$ (Product of the action of heparinases II and III on heparin and heparan sulphate)
ΔUA → 2S-Glc*N*Ac Na_2 (III-A)	$C_{14}H_{19}NO_{14}SNa_2$ (Minor product of the action of heparinase II on heparin)
ΔUA → Glc*N*Ac Na (IV-A)	$C_{14}H_{20}NO_{11}Na$ (Produced from heparin sulphate by digestion With heparinase III)
ΔUA → Gal*N*Ac-4S Na_2 (ΔDi-4S)	$C_{14}H_{19}NO_{14}SNa_2$ (Produced from various chondroitin sulphates By the action of chondroitinases ABC, B and AC-1)

ΔUA → Gal*N*Ac-6S Na2 (ΔDi-6S)	$C_{14}H_{19}NO_{14}SNa_2$ (Produced from various chondroitin sulphates By the action of chondroitinases ABC, AC-1 and C)
ΔUA → Gal*N*Ac-4S,6S Na_3 (ΔDi-disE)	$C_{14}H_{18}NO_{17}S_2Na_3$ (Produced from various chondroitin sulphates By the action of chondroitinases ABC, B and AC-1)
ΔUA → 2S-Gal*N*Ac-4S Na_2 (ΔDi-disB)	$C_{14}H_{18}NO_{17}S_2Na_3$ (Produced from various chondroitin sulphates by action of chondroitinase ABC and/or B. Most typically from chondroitin sulphate B (dermatan sulphate))
ΔUA → 2S-Gal*N*Ac-6S Na_3 (ΔDi-disD)	$C_{14}H_{18}NO_{17}S_2Na_3$ (Produced from various chondroitin sulphates by the action of chondroitinase ABC)
ΔUA → 2S-Gal*N*Ac-4S-6S Na_4 (ΔDi-tisS)	$C_{14}H_{17}NO_{20}S_3Na_4$ (Produced as a minor component by the action of chondroitinase ABC on various chondroitin sulphates, particularly B)
ΔUA → 2S-Gal*N*Ac-6S Na_2 (ΔDi-UA2S)	$C_{14}H_{19}NO_{14}SNa_2$ (Produced as a minor component from various chondroitin sulphates by the action of chondroitinase ABC)
ΔUA → Glc*N*Ac Na (ΔDi-HA)	$C_{14}H_{20}NO_{11}Na$ (The only unsaturated disaccharide produced from hyaluronic acid by the action of chondroitinase ABC or AC-1)
Hyaluronan fragments (4mer)	(GlcAβ1-3Glc*N*Acβ1-4)n (n=4)
Hyaluronan fragments (8mer)	(GlcAβ1-3Glc*N*Acβ1-4)n (n=8)
Hyaluronan fragments (10mer)	(GlcAβ1-3Glc*N*Acβ1-4)n (n=10)
Hyaluronan fragments (12mer)	(GlcAβ1-3Glc*N*Acβ1-4)n (n=12)
Heparin	(GlcA/IdoAα/β1-4Glc*N*Acα1-4)n (n=200)
Chondroitin sulfate	(GlcA/IdoAβ1-3(±4/6S)Gal*N*Acβ1-4)n (n<250)
Dermatan sulfate	((±2S)GlcA/IdoAα/b1-3(±4S)Gal*N*Acβ1-4)n (n<250)
Chondroitin 6-Sulfate	(GlcA/IdoAβ1-3(±6S)Gal*N*Acβ1-4)n (n<250)
HA - 4	(GlcAβ1-3Glc*N*Acβ1-4)n (n=4)
HA - 6	(GlcAβ1-3Glc*N*Acβ1-4)n (n=6)
HA - 8	(GlcAβ1-3Glc*N*Acβ1-4)n (n=8)
HA 10	(GlcAβ1-3Glc*N*Acβ1-4)n (n=10)
HA-12	(GlcAβ1-3Glc*N*Acβ1-4)n (n=12)
HA-14	(GlcAβ1-3Glc*N*Acβ1-4)n (n=14)
HA-16	(GlcAβ1-3Glc*N*Acβ1-4)n (n=16)
HA 30000 Da	(GlcAβ1-3Glc*N*Acβ1-4)n
HA 107000 Da	(GlcAβ1-3Glc*N*Acβ1-4)n
