# Supplementary material for: The dCache Chemoreceptor TlpA of Helicobacter pylori Binds Multiple Attractant and Antagonistic Ligands via Distinct Sites
Source: mBio. 2021 Aug 3;12(4):e01819-21. doi: 10.1128/mBio.01819-21 (PMC8406319; doi:10.1128/mBio.01819-21)
Supplement: TABLE S2 [file mbio.01819-21-st002.pdf]

Cluster	Amino acids
**A**	**PHE-203**, ILE-205, VAL-211, ILE-224, **TYR-228**, VAL-231, ALA-234, THR-235, VAL-238, LEU-250, TYR-252, LEU-263, VAL-265, ALA-285, **ILE-287**
B	GLY-206, VAL-207, LYS-208, LEU-242, GLU-243, LYS-269, LEU-280, ASN-281
C	VAL-60, ASN-63, THR-64, SER-67, ALA-94, ASN-95, SER-96, HIS-97
**D**	SER-102, MSE-103, PHE-104, THR-114, GLU-126, ASN-133, **ALA-136**, **ARG-153,** **TYR-151**, LEU-167, ALA-181, LEU-182, MET-183
E	PHE-105, LYS-106, ASN-107, ARG-108, ASN-172, VAL-179
F	LYS-138, ASN-142, GLU-144, ILE-145, SER-146, SER-276, LYS-277, ASP-278
G	PRO-76, LYS-77, ASP-78, THR-79, LYS-84, PHE-105, ASN-107, ARG-108, LEU-111, ASN-172, GLU-173, VAL-179
H	SER-96, HIS-97, VAL-98, ALA-99, ARG-117, ASP-118, MSE-155, PRO-156, ASN-157, ALA-159, VAL-161, SER-187, SER-190
