# Supplementary material for: The dCache Chemoreceptor TlpA of Helicobacter pylori Binds Multiple Attractant and Antagonistic Ligands via Distinct Sites
Source: mBio. 2021 Aug 3;12(4):e01819-21. doi: 10.1128/mBio.01819-21 (PMC8406319; doi:10.1128/mBio.01819-21)
Supplement: TABLE S3 [file mbio.01819-21-st003.pdf]

	**Fumarate**	**Arginine**
**Cluster**	Cluster Occupation %	Cluster Occupation %
**A (membrane-proximal Cache)**	**45**	25
B	10	5
C	20	5
**D (membrane-distal Cache)**	10	**55**
E	5	-
F	10	-
G	-	5
H	-	5
