# Supplementary material for: The dCache Chemoreceptor TlpA of Helicobacter pylori Binds Multiple Attractant and Antagonistic Ligands via Distinct Sites
Source: mBio. 2021 Aug 3;12(4):e01819-21. doi: 10.1128/mBio.01819-21 (PMC8406319; doi:10.1128/mBio.01819-21)
Supplement: TABLE S4 [file mbio.01819-21-st004.pdf]

**Primer name**	**Sequence (5’ → 3’)**
TlpA_D165A_For	GGGGCGGAAGTTTATGGAGTT**GCT**ATTCTTTTACCTTTATTG
TlpA_D165A_Rev	CAATAAAGGTAAAAGAAT**AGC**AACTCCATAAACTTCCGCCCC
TlpA_M183A_For	GAGGTTGTAGGGGCTTTG**GCG**GTTTTTATTTCCATTGACAGC
TlpA_M183A_Rev	GCTGTCAATGGAAATAAAAAC**CGC**CAAAGCCCCTACAACCTC
TlpA_Y228A_For	GACAAACCTATCGCAGAAATT**GCT**AAGAGCGTACCTAAAGCC
TlpA_Y228A_Rev	GGCTTTAGGTACGCTCTT**AGC**AATTTCTGCGATAGGTTTGTC
TlpA_Y252A_For	CTCTAAAGCGACTTTAGAA**GCT**TTAGATCCCTTTAGCCATAAGG
TlpA_Y252A_Rev	CCTTATGGCTAAAGGGATCTAA**AGC**TTCTAAAGTCGCTTTAGAG
TlpA_D254A_For	CTAAAGCGACTCTAGAATACTTA**GCT**CCCTTTAGCCATAAGG
TlpA_D254A_Rev	CCTTATGGCTAAAGGG**AGC**TAAGTATTCTAGAGTCGCTTTAG
