# Supplementary material for: The dCache Chemoreceptor TlpA of Helicobacter pylori Binds Multiple Attractant and Antagonistic Ligands via Distinct Sites
Source: mBio. 2021 Aug 3;12(4):e01819-21. doi: 10.1128/mBio.01819-21 (PMC8406319; doi:10.1128/mBio.01819-21)
Supplement: TABLE S5 [file mbio.01819-21-st005.pdf]

Classification	Guidelines
**1. Sample: Glycan Binding Sample**	
Description of Sample	<u>Sample names:</u><br>TlpA protein<br><u>Origin:</u> *Helicobacter pylori* SS1*;* produced as a recombinant His -tagged protein in *E. coli*.<br><br><u>Method of preparation:</u><br>The preparation of TlpA is explained in the Materials and Methods section: TlpA construct design and protein purification.
Sample modifications	Sample is a His Tagged protein containing only the periplasmic receptor domain, amino acids 28-299, of the full length TlpA transmembrane protein.
Assay protocol	**Please see *Materials and Methods:* Ligand binding array*.***<br><br>Proteins were complexed with mouse anti-his antibody, with secondary and tertiary AlexaFluor555 antibodies (rabbit anti-mouse, goat anti-rabbit) at a molar ratio of 4:2:1. The protein/antibody complex was incubated on ice for 10 mins prior to placing on the array. Arrays were performed with 1 µg of complexed protein incubated on the array for 20 min. Arrays were then washed 3 times in Array PBS (PBS + 2 mM $MgCl_2$ + 2 mM $CaCl_2$) then dried by centrifugation for 3 min at 300 x *g*. Scanning was performed using an Innopsys InnoScan 1100AL and analyzed using Mapix (Innopsys). Yes/no binding was determined by six positive replicate spots in three replicate experiments. Positive binding was determined by spots being significantly greater than negative control spots by two-tailed *t* test.
**2.1 Glycan Library**	
Glycan description for defined glycans	Glycans in this study are listed in Table S1 and is a published library (Waespy et al., 2015).
Glycan description for undefined glycans	N/A.
Glycan modifications	Glycans were prepared in one of two ways for printing:<br>1. Glycans (with IDs in number/letter format; e.g. 1A, 4C, 7K) were sourced commercially from Dextra Laboratories, Elicityl and Carbosythn and were made into glycoamines using a published protocol (Day et al., 2009).<br>2. Glycans (with IDs in number only format) were obtained from Prof. Nicolai Bovin and were modified with spacers (Blixt et al., 2004). The library of these glycans was first published in (Huflejt et al., 2009).
**2.2 Small molecule Library**	
Sample description for defined compounds	Compounds printed in the amino acid/small molecule array for this study are listed in Table S1.

3. Printing Surface; e.g., Microarray Slide	
Description of surface	Epoxy activated glass microarray slides.
Manufacturer	ArrayIt SuperEpoxy 3 (SME3).
Custom preparation of surface	N/A.
Non-covalent Immobilisation	N/A.
**4. Arrayer (Printer)**	
Description of Arrayer	**Glycan array:** SpotBot® Extreme Protein Microarray Spotter (ArrayIt, California, USA).<br>**Small molecule array:** ArrayJet Argus Marathon non-contact printer.
Dispensing mechanism	**Glycan array:** Contact printing using 946NS6 pins with a 6 pin in a 3 columns x 2 rows configuration.<br>**Small molecule array:** Non-contact jet printing
Glycan and small molecule deposition	**Glycan array:** Approximately 1.8 nl per spot is printed according to manufactures guidelines.<br>Glycan were at 500 µM in 50:50 DMF:DMSO.<br>**Small molecule array:** Approximately 320 nl per spot is printed according to manufactures guidelines.<br>Samples were at 1000 µM in PBS with 2% glycerol
Printing conditions	**Glycan array:** Array were printed with dehumidification at a maximum humidity of 60% relative humidity (Standard laboratory starting humidity of 75-90%) at 22ºC. Glycans were left to react with the slide for at least 8 hours after the print was completed.<br>**Small molecule array:** Array were printed with dehumidification at a maximum humidity of 60% relative humidity (Standard laboratory starting humidity of 75-90%) at 22ºC. Samples were left to react with the slide for at least 15 hours after the print was completed.
**5.1 Glycan array with "Map"**	
Array layout	The array consists of a single array of glycans split between 6 pins (3 columns x 2 rows) with 4500 µm row and column spacing. Each pin printed a 20 columns x 16 rows with 200 µm spot spacing (center to center) with a minimum spot size of 100µm. Each sample is printed in quadruplicate with each of the 6 print areas including at least three negative control samples (print solution only) and two positive control samples consisting of one sample of fluoroscienamine and one sample of a mixture of rabbit anti-mouse antibody labeled with Alexa 555 and Alexa 647. Positive controls provide proof of successful immobilization of the amine reagents and provides for orientation for analysis. The antibodies also can provide controls for secondary antibodies used in experiments (if applicable).
Glycan identification and quality control	Arrays are quality controlled by a range of measures. 1. Each printed array is post print scanned to confirm deposition of the glycans on the array surface prior to neutralization of the remaining slide surface. 2. Post neutralized slides are scanned again to monitor for remaining autofluorescence. 3. Slides are assayed with fluorescently labeled lectins: WGA-Texas Red (EY Laboratories) and ConA-FITC (EY Laboratories).

5.2 Small molecule array with “Map”	
Array layout	The array consists of triplicate sub-arrays of amino acids and small moleucles (24 rows x 10 columns) with 400µm spot spacing (centre to centre) with a minimum spot size of 100µm. Each sample is printed six times, with each of the 3 sub-arrays including at least six negative control samples (print solution only) and a positive control samples consisting of one a mixture of rabbit anti-mouse antibody labeled with Alexa 555 and Alexa 647. Positive controls provide proof of successful immobilization of the amine reagents and provides for orientation for analysis.
Sample identification and quality control	Arrays are quality controlled by a range of measures. 1. Each printed array is post print scanned to confirm deposition of the samples on the array surface prior to neutralization of the remaining slide surface. 2. Post neutralized slides are scanned again to monitor for remaining autofluorescence.
**6. Detector and Data Processing**	
Scanning hardware	Inopsys InnoScan 1100AL (Lasers: 488 nM, 532 nM with two filter sets for analysis at 532 and 595 nM), 635 nM) scanner.
Scanner settings	Scanning resolution: 10 µM<br>Laser channel: 532 nM operating 595 nM excitation / 625 nM emission filter set.<br>PMT: 20% gain<br>Scan powers: Low laser power.
Image analysis software	Innopsys MAPIX.
Data processing	Data was exported as a CSV file and exported to Microsoft Excel.
**7. Glycan Microarray Data Presentation**	
Data presentation	Glycan array: Data is presented as yes/no binding in Table 1. The full list of glycans is shown in Table S1.
**8. Interpretation and Conclusion from Microarray Data**	
Data interpretation	**Glycan arrays:** We only use glycan arrays as a yes/no binding tool. Due to this we look only at binding that is unambiguously above background vs lack of binding above background. Average background + 3x standard deviation of the background of 20 sets of 4 spots of DMF:DMSO only spots is applied to determine if binding observed is significantly above background. Only spots with values equal to or greater than this value were considered as binding from data of any tested slide. These values are slide dependent.<br>**Small molecule arrays:** We only use amino acid arrays as a yes/no binding tool. Due to this we look only at binding that is unambiguously above background vs lack of binding above background. Average background + 3x standard deviation of the background of 6 spots of PBS + 2% glycerol only spots is applied to determine if binding observed is significantly above background. Only spots with values equal to or greater than this value were considered as binding from data of any tested slide. These values are slide dependent.
Conclusions	TlpA binds multiple ligands. Subsequent work showed that some of these are attractants, some elicit no response, and some are antagonists.
